# Expanded Functionality and Portability for the Colvars Library

**DOI:** 10.1021/acs.jpcb.4c05604

**Published:** 2024-11-06

**Authors:** Giacomo Fiorin, Fabrizio Marinelli, Lucy R. Forrest, Haochuan Chen, Christophe Chipot, Axel Kohlmeyer, Hubert Santuz, Jérôme Hénin

**Affiliations:** † National Institute of Neurological Disorders and Stroke, Bethesda, Maryland 20814, United States; ‡ National Heart, Lung and Blood Institute, Bethesda, Maryland 20892, United States; § Department of Biophysics and Data Science Institute, Medical College of Wisconsin, Milwaukee, Wisconsin 53226-3548, United States; ∥ Theoretical and Computational Biophysics Group, Beckman Institute, and Department of Physics, University of Illinois at Urbana−Champaign, Urbana, Illinois 61820, United States; ⊥ Laboratoire International Associé CNRS et University of Illinois at Urbana−Champaign, UMR 7019, Université de Lorraine, 54506 Vandœuvre-lès-Nancy, France; # Department of Biochemistry and Molecular Biology, The University of Chicago, 929 E. 57th Street W225, Chicago, Illinois 60637, United States; ∇ Department of Chemistry, The University of Hawai’i at Manoa, 2545 McCarthy Mall, Honolulu, Hawaii 96822, United States; ○ Institute for Computational Molecular Science, Temple University, Philadelphia, Pennsylvania 19122, United States; ◆ Laboratoire de Biochimie Théorique UPR 9080, Université Paris Cité, CNRS, 75005 Paris, France

## Abstract

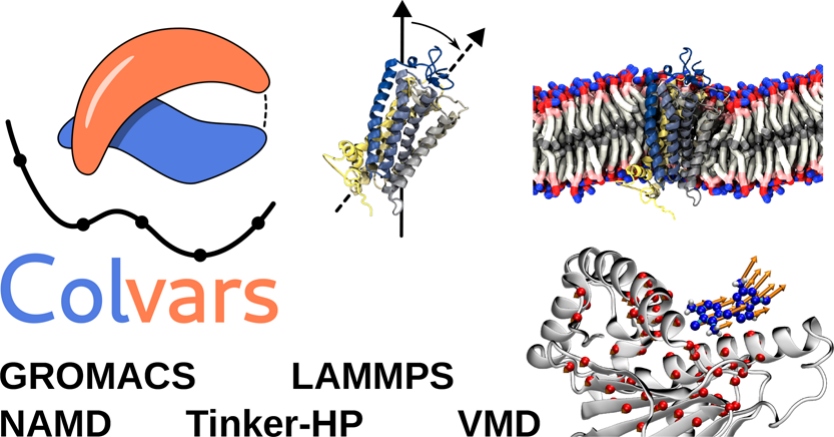

Colvars is an open-source C++ library that provides a modular toolkit for collective-variable-based molecular simulations. It allows practitioners to easily create and implement descriptors that best fit a process of interest and to apply a wide range of biasing algorithms in collective variable space. This paper reviews several features and improvements to Colvars that were added since its original introduction. Special attention is given to contributions that significantly expanded the capabilities of this software or its distribution with major MD simulation packages. Collective variables can now be optimized either manually or by machine-learning methods, and the space of descriptors can be explored interactively using the graphical interface included in VMD. Beyond the spatial coordinates of individual molecules, Colvars can now apply biasing forces to mesoscale structures and alchemical degrees of freedom and perform simulations guided by experimental data within ensemble averages or probability distributions. It also features advanced computational schemes to boost the accuracy, robustness, and general applicability of simulation methods, including extended-system and multiple-walker adaptive biasing force, boundary conditions for metadynamics, replica exchange with biasing potentials, and adiabatic bias molecular dynamics. The library is made available directly within the main distributions of the academic software GROMACS, LAMMPS, NAMD, Tinker-HP, and VMD. The robustness of the software and the reliability of the results are ensured through the use of continuous integration with a test suite within the source repository.

## Introduction

In the field of molecular simulations, projecting high-dimensional configurations into a low-dimension space of collective variables (CVs) is a common way to perform enhanced sampling,^1^ and a nearly universal tool for analyzing simulation data to extract physical, chemical, and biological insight.

Computational techniques, including CV-based ones, are generally useful insofar as a reliable and efficient software implementation exists for them. Accordingly, the most commonly used simulation methods in molecular simulation (interatomic potentials, thermostats, barostats, multiple time-stepping) have gained wide distribution via software applications that could be installed directly by their intended users. However, methods for CV-based enhanced sampling come in a broader variety compared to those for conventional MD simulations, a fact that hinders their bottom-up reimplementation in each software package. To address this issue, we have previously introduced the collective variables module (Colvars), a software library designed for distribution with multiple simulation and analysis software packages.^2^

The close integration between Colvars and each package has greatly simplified many computational protocols, to the extent that Colvars has been perceived in a few cases to be an exclusive feature of NAMD,^3^ the package to which Colvars was first added. It is more accurate instead to consider Colvars as distinct from the CV implementations specific to each MD package, such as the pull code of GROMACS^4^ or the CustomCVForce feature of OpenMM,^5^ as well as from independently distributed plugins such as PLUMED.^6^

Further customization is available through a scripting interface, or a graphical user interface (GUI)^7^ within VMD.^8^ Additionally, the Colvars library is under continuous development, with many of its features being introduced over multiple major releases of the packages with which it is distributed. Therefore, it is useful to review the main extensions and new features brought to the library since the original publication.^2^

Following a brief summary of the underlying theoretical concepts, this manuscript reviews new or improved collective variables to reconstruct the energy landscape of multiple macromolecules in contact with each other as well as their own conformational changes. Also reviewed are the improvements to established sampling schemes such as adaptive biasing force (ABF) and metadynamics, and the introduction of new schemes to bias the simulated ensemble toward experimental data. Lastly, the enhanced interfaces to the NAMD and LAMMPS^9^ packages, as well as the interfaces to VMD, Tinker-HP and GROMACS are also discussed.

## General Architecture of Colvars

A collective variable, often abbreviated as “colvar” or CV, can be any function ξ(**X**, **u**) of the Cartesian coordinates of multiple atoms **X** and the periodic cell parameters **u**, such that the values of ξ map physically relevant regions of configurational space. To enhance the sampling of rare states in MD simulations, external forces are added in colvar space and propagated to the equations of motion of the individual atoms in Cartesian space. Owing to the generality of this scheme, any mathematical function can define a CV, as long as it is continuous and differentiable in all its arguments.

The most common use of Colvars is a continuous simulation of a single copy of the model system, under the effect of external forces whose effects are measured and included in the estimate of a free-energy profile for the system at equilibrium. It is, however, also straightforward to use Colvars with methods that introduce artificial discontinuities in the atomic trajectory^10^ or rely on selecting simulation snapshots without applying external biasing forces,^11−14^ or other multicopy simulations.^15^

As with conventional MD simulations, computation using methods implemented by Colvars requires the user to define two entities:

- At least one collective variable, by selecting atoms and a function of those atoms’ coordinates: this is a generalization of interatomic variables such as bonds, angles, and dihedrals.
- A scheme to govern the dynamics of the colvar just defined: this is a generalization of interatomic potentials; however, because the typical use is to achieve biased sampling, this scheme is called a “*bias*”.

Multiple variables and biases can be simultaneously defined. The Colvars library offers many choices of definition for each, ranging from massively parallel, compiled code to scripted functions defined by the user at run time; both are described below. This fully modular architecture gives practitioners access to a broad space of possible collective variables and types of biased dynamics.

## Notable or New Coordinates

### Euler and Polar Angles

**Figure 1:**
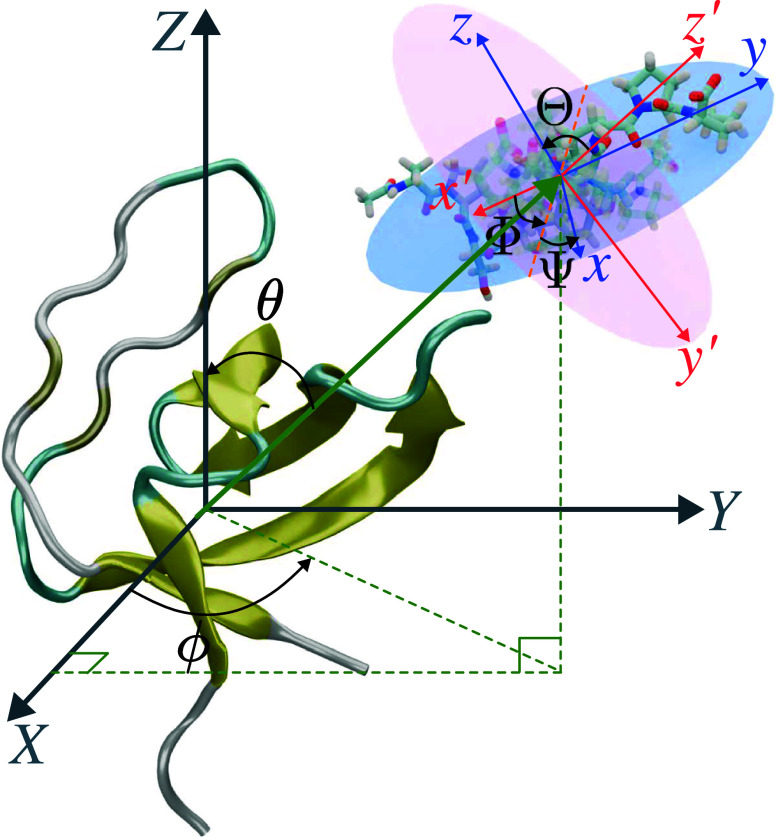
Schematic representation of the polar angles (ϕ, θ) and the Euler angles (Φ, Θ, Ψ) in protein–ligand binding. The polar angles (ϕ, θ) describe how the ligand revolves around the protein. The Euler angles (Φ, Θ, Ψ) depict the rotation of the ligand around its origin.

To facilitate the description of the spatial arrangement of a ligand and a protein as one-dimensional variables in binding free-energy calculations,^16^ the Colvars library has introduced (i) the polar (θ) and azimuthal (ϕ) angles that describe the position of an atom group in spherical coordinates,^17^ and (ii) the “roll” (Φ), “pitch” (Θ) and “yaw” (Ψ) angles that depict the rotation of an atom group with respect to a reference frame.^18^ These angles are calculated as
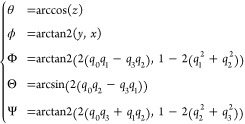
where (*x*, *y*, *z*) is the unit vector from the origin to the center-of-mass of the molecule, and (*q*_0_, *q*_1_, *q*_2_, *q*_3_) is the quaternion describing the rotation that minimizes the root-mean-square-deviation (RMSD) of the molecule with respect to the reference.^2,19^ A schematic representation of these angles is shown in Figure 1. The radial distance *r*, the angles θ and ϕ form a complete set of polar coordinates, which is defined in Colvars using the distance, polarTheta, and polarPhi keywords. Conversely, the angles Φ, Θ and Ψ are defined using the eulerPhi, eulerPsi and eulerTheta keywords, which define internally the computation of a quaternion **q** from which the relevant projection is carried out. The set of functions described above is extensively used by the Binding Free Energy Estimator.^20,21^ Like any other collective variable, these angles can be computed in a moving frame of reference tied to a specified group of atoms (see section below).

### Path Collective Variables

When considering a pathway that connects two metastable states, A and B, of the free-energy landscape underlying a geometric transformation—e.g., the conformational transition of a protein—it becomes advantageous to associate a progress variable^22,23^ to this pathway, which can be supplied, for instance, by path-optimization strategies like the string method and its variants,^24−26^ or, more recently, by machine-learning strategies aimed at discovering the committor, i.e., the probability that, starting from a given configuration, the target state, B, will be reached before returning to the reference state, A.^27−29^ Such an approach facilitates the calculation of the free-energy change between the reference and the target states. The concept of a progress variable also offers a robust framework for reducing dimensionality,^30−32^ while providing a concise description of potentially complex geometric transformations by means of a one-dimensional free-energy profile, or “potential of mean force”. The latter ideally captures the dynamics of the molecular processes at play in the transition between the two end-states.

Computing a progress variable necessitates the projection of the collective variable, or alternatively, the Cartesian-coordinate space onto the path representing the average transition, resulting in differentiable expressions. These expressions characterize a continuous pathway along which a free-energy change can be precisely determined. An example of such expressions is furnished by the so-called path-collective variables (PCVs), which are introduced here in a variant of their original arithmetic formulation,^33^
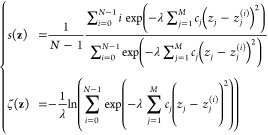
1where *c*_*j*_ is the weight of the *j*th CV, *z*_*j*_^(*i*)^, the value of this CV for the *i*th reference frame, or node of the pathway, and *z*_*j*_, the value of *j*th CV for the current frame. *M* and *N* are the number of CVs and the number of reference frames, respectively. λ serves as a smoothing parameter that relates to the inverse of the mean squared displacement between consecutive images. Variable *s*(**z**) acts as a progress parameter along the pathway, ranging from 0 (state A) to 1 (state B), while the ancillary variable ζ(**z**) can be interpreted as the radius of a tube enveloping the pathway, confining the sampling within its vicinity. This conceptual framework aids in determining the underlying free energy profile, most notably from a coarse approximation of the pathway, by mapping the free energy in the two dimensions, *s*(**z**) and ζ(**z**), denoted aspathCV and azpathCV in the Colvars library. The one-dimensional free-energy profile is then derived from the marginal distribution of *s*(**z**).

An alternative to the previously stated definition of the PCV is provided by the original arithmetic expression of Branduardi et al.,^33^ resting on mean squared displacements between the current position in Cartesian-coordinate space and that of the images of the string. These PCV are called aspath and azpath in the Colvars library, and have proven useful when it is not possible to infer, primarily from human intuition, the subspace of CV needed to optimize the pathway.

While the free energy should ideally remain immune to changes in λ for straightforward linear abscissas, the complexity of the CV or Cartesian space, along with the nonlinearity of the string, complicates its choice and is prone to induce instabilities in the trajectory due to the singularity of the mean squared displacement as it approaches zero. This ailment can be alleviated by means of a normalized exponential function (Softmax). Still, even minor deviations from the optimal value of λ may result not only in sampling inefficiencies but also in discernible artifacts that compromise the physical accuracy of sampling. These limitations have led to the development of alternative geometric expressions that are more robust to parameter selection. For example, path-metadynamics variables (PMVs) generate unique values for *s*(**z**) and ζ(**z**), unlike the original arithmetic PCV formulation, and are defined as follows,^34^
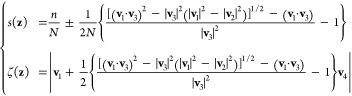
2Here, **v**_1_ = **s**_*n*_ – **z** is a vector pointing from the current position to the nearest image, **v**_2_ = **z** – **s**_*n*–1_, a vector pointing from the second nearest image to the current position, **v**_3_ = **s**_*n*+1_ – **s**_*n*_, a vector pointing from the nearest to the third nearest image, and **v**_4_ = **s**_*n*_ – **s**_*n*–1_, a vector pointing from the second nearest to the nearest image. Here, *n* is the index of the nearest image, and *N* is the total number of images. If the current position is "to the left" of the nearest reference image, the sign in the expression of *s*(**z**) is positive—otherwise, it is negative. The above PMV, *s*(**z**) and ζ(**z**), are denoted gspathCV and gzpathCV in the Colvars library, and, just like the PCV, possess a related definition in Cartesian-coordinate space, referred to as gspath and gzpath.

### Moving Frame of Reference

Many applications of Colvars are focused on studying changes in structure within one macromolecule, or the interactions between multiple macromolecules. However, in some of these cases, the CVs employed are sensitive to translations or rotations of those macromolecules, making the associated free-energy landscape unnecessarily complex. To address this issue, Colvars allows for any CVs to be defined in an invariant frame of reference, whose axes and point of origin follow the motion of a chosen group of atoms. The same methodology^19^ and implementation^2^ used in the computation of optimal RMSDs also underlie the definition of such a frame of reference.

A typical example of this feature is a protein/ligand complex, where the protein defines the moving frame of reference, and the ligand’s movements are parametrized by specific CVs expressed in that frame. These CVs may include, for example, the translations and Euler angles used in the Binding Free-Energy Estimator (BFEE) protocol,^18,35^ or the RMSD from a reference structure in the Distance from Bound Configuration (DBC) coordinate used in the SAFEP approach.^36,37^ Notably, the implementation places no restrictions on the number of frames of reference that may be defined concurrently and is sufficiently modular to support arbitrary types of CVs. For example, a moving frame of reference may be combined with a symmetry-invariant RMSD coordinate for efficient restraining of symmetric ligands.^38^

A moving frame of reference is defined by the fittingGroup keyword, which selects the set of atoms whose frame of reference a CV may be defined on. The set of fitting atoms is defined separately from the atoms explicitly used in defining a CV, and the coordinates of the latter set of atoms are transformed into the moving frame whenever the CV is being recomputed. In mathematical terms any function ζ(**X**) may be defined in a moving frame as
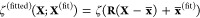
3where **x̅** is the center of geometry of **X**, **R** is the optimal rotation matrix, **X**^(fit)^ are the Cartesian coordinates of the fitting atoms, **x̅**^(fit)^ their center of the geometry. Because *R* is a function of **X**^(fit)^, any biasing force applied to ζ^(fitted)^ is also propagated to the fitting atoms by implicitly calculating the gradients of ζ^(fitted)^ with respect to **X**^(fit)^ as detailed in the Supporting Information.

While this treatment allows to consistently bias all degrees of freedom associated with ζ^(fitted)^, the additional computation requires nested loops over both **X** and **X**^(fit)^, which may be computationally costly. Such consideration applies to all moving frames of reference, with the exception of the special case when **X** and **X**^(fit)^ are the same coordinates and ζ is the RMSD function, for which the fitting gradients are zero by definition and are therefore not computed. In all other cases, it is good practice to define a CV and a moving frame of reference to minimize the number of atoms **X** selected for the CV and the fitting atoms **X**^(fit)^, or their product.

### DEER CVs

**Figure 2:**
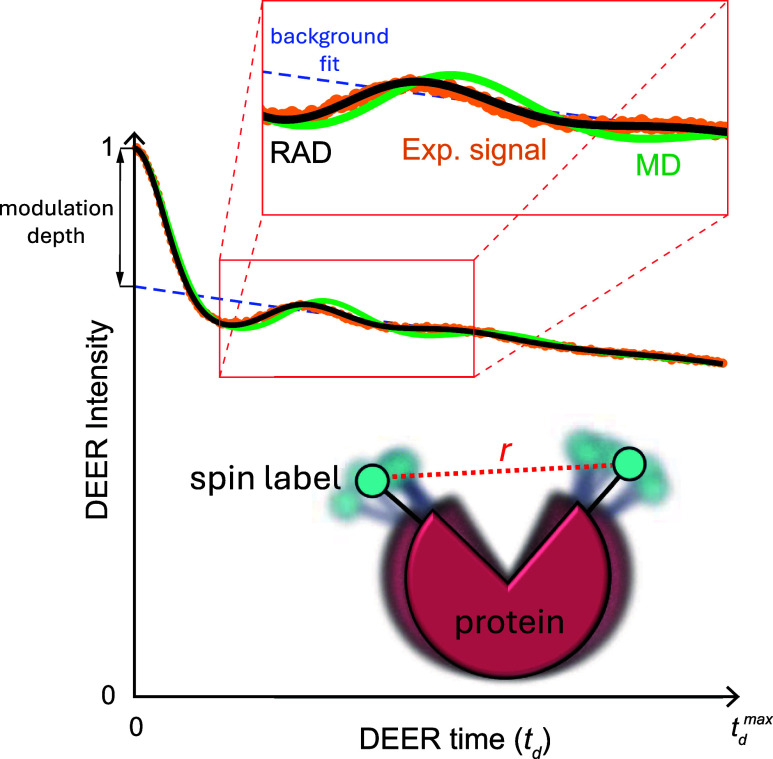
Example of experimental and calculated DEER time-domain signals. Also shown below the DEER traces is a schematic of the protein with attached spin labels. The experimental signal (orange circles) is from DEER measurements on spin-labeled T4 Lysozyme at positions 62 and 109.^39−41^ DEER traces calculated using conventional MD and the restrained average dynamics (RAD) technique^42^ are shown as green and black lines, respectively, representing the time average of the deer CV (see eq 4). The blue dashed line represents the fit of the background function, (1 – Λ_1_) exp–(Λ_2_|*t*_d_|), at large times when oscillations are minimal.

A primary objective of molecular dynamics simulations is to provide a molecular interpretation of complex experimental signals, particularly those highlighting large-scale conformational changes in biomolecules, like double electron–electron resonance (DEER) measurements. This technique measures long distances (up to 16 nm^43^) between spin labels on a biomolecule (Figure 2). To compute the DEER signal of a biomolecule with two attached spin labels, we implemented a vector-valued CV in Colvars, named deer. This CV assesses the contribution of an individual biomolecular configuration to the signal.

The implementation of the deer CV is based on the standard approximation of nearly isolated spin pairs in a dilute solution,^44,45^ enabling the deer CV to be expressed as a function of the spin labels’ distance, *r* (the distance between nitroxide groups in MTSSL spin labels):
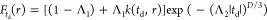
4where *t*_d_ denotes the time of the DEER signal, spanning an interval with length provided as an input parameter. Each *t*_d_ value corresponds to a specific vector component of the deer CV. The experimental parameters Λ_1_ and Λ_2_ characterize the signal modulation depth and the background contribution from spin–spin interactions between different biomolecules in the solution, respectively (Figure 2). The parameter *D* describes the system dimensionality (3 for a solvated biomolecule in a homogeneous sample and 2 for a membrane-embedded biomolecular system). The function *k*(*t*_*d*_, *r*) (DEER kernel) represents the intramolecular spin–spin contribution to the signal and is the only term in eq 4 that depends on the molecular configuration via the spin labels’ distance, *r*. The analytical expression for this term, based on the same approximations and assuming ideal pulses, is given by^46−48^
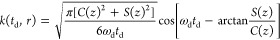
5where ω_d_ = *g*^2^μ_B_^2^μ_0_/4π*ℏr*^3^ denotes the dipolar frequency.  and  are the cosine and sine Fresnel integrals ().

Once the deer CV is evaluated along the MD trajectory, the predicted DEER signal can be calculated as the time average of the CV: ⟨*F*_*t*_d__(*r*) ⟩ = [(1 – Λ_1_) + Λ_1_⟨*k*(*t*_d_, *r*)⟩] exp[–(Λ_2_|*t*_d_|)^*D*/3^] (where ⟨··· ⟩ denotes ensemble average). This can be computed, for example, using the running average functionality in Colvars or a custom script. The experimental parameters, Λ_1_ and Λ_2_, can be provided in the input if they are known a priori (e.g., through background fitting as shown in Figure 2). Alternatively, we also implemented routines in Colvars to obtain these parameters automatically by best-fitting predicted and experimental signals.^42^ The current implementation also supports the use of only the DEER kernel function in eq 5, referred to as the deerkernel CV. This can provide a prediction of the background-corrected and shifted DEER signal (⟨*k*(*t*_d_, *r*)⟩), which can be inferred from the measured signal if Λ_1_ and Λ_2_ are known.

Overall, the deer and deerKernel CVs enable comparison of predicted and experimental DEER signals for rigorous molecular interpretation. They can also be used in combination with refinement methods like Restrained Average Dynamics (RAD)^42^ to align simulation ensembles with experimental data (see the section on biasing methods below). At the time of writing, these feature are available in a Colvars development branch and are slated for integration into the standard release.

### Machine-Learned CVs

**Figure 3:**
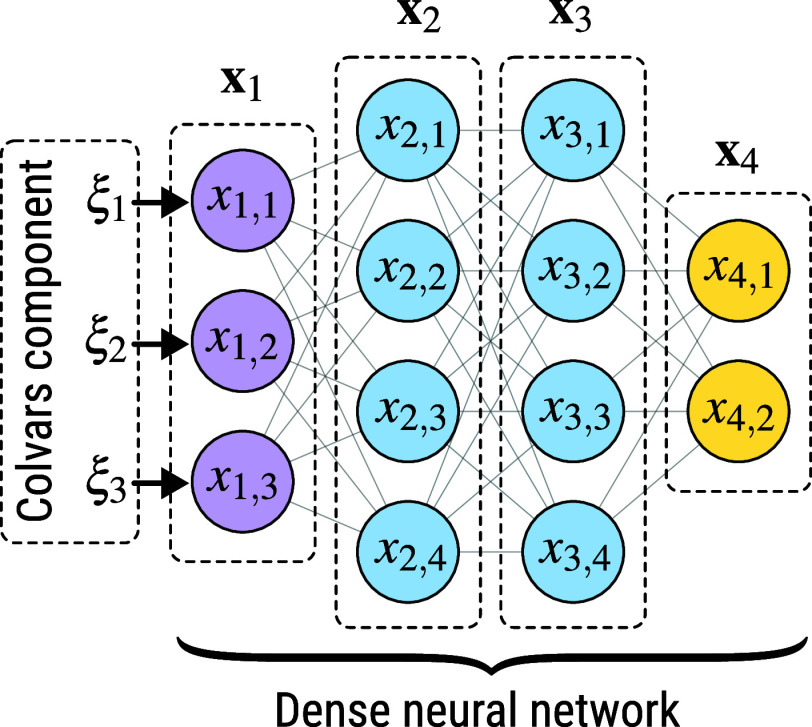
Schematic representation of a dense neural network. Each circle represents a node, and nodes within the input, hidden, and output layers are marked in purple, blue, and yellow, respectively.

In principle, according to the universal approximation theorem,^49^ a neural network (NN) with hidden layers can be used to approximate any continuous function. Thanks to this advantage, machine learning using NNs has become a widely used strategy for dimensionality reduction and discovering CVs.^50−60^ To make use of these CVs in molecular simulations, we have implemented in Colvars the machine-learned CV (MLCV) component,^58^ referred to as neuralNetwork. This function can use any of the available CVs available in Colvars as inputs, and forward them into a user-defined dense neural network (NN): the output of the network’s last layer provides the value for the CV. In a dense NN, the output of the *j*-th node at the *k*-th layer that has *N*_*k*_ nodes is computed as
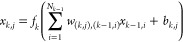
6where *f*_*k*_ is the activation function of the *k*th layer, *w*_(*k*,*j*),(*k*–1,*i*)_ is the weight of the *j*th node at the *k*th layer with respect to the *i*th node at the (*k* – 1)th layer, *b*_*k*,*j*_ is the bias of the *j*th node at the *k*-th layer, and *N*_*k*–1_ is the number of nodes at the (*k* – 1)th layer. The computation can be also written in the vector form **x**_*k*_ = **f**_*k*_(**W**_*k*,*k*–1_**x**_*k*–1_ + **b**_*k*_), where **f**_*k*_ is an *N*_*k*_-dimension activation function, **W**_*k*,*k*–1_ is an *N*_*k*_ × *N*_*k*–1_ weight matrix, **x**_*k*–1_ is the output of the previous layer, and **b**_*k*_ is an *N*_*k*_-dimension bias vector. A schematic representation of the structure of a neural network can be found in Figure 3. To make the MLCV agnostic of the underlying deep-learning frameworks, the weights and biases of the dense NN are provided in plain text files, and the activation functions can be defined using the Lepton library.^61^ The MLCV has been used, for instance, to implement the CVs discovered by autoencoders for describing the folding of proteins.^62^

In addition to the neuralNetwork feature, which is available directly within the Colvars library, more general NN coordinates can be computed through an interface to the PyTorch library. This functionality, available through the torchANN keyword, allows for computing colvars as functions of existing variables defined by a NN in PyTorch format. The ability to use the torchANN feature is conditional upon the presence of a Torch library linked to the MD engine, or dynamically loaded at runtime.

### Volumetric Map-Based CVs

Traditionally, biased MD simulations approaches have often been used to simulate changes in conformation of few individual molecules, such as proteins or nucleic acids. However, supra-molecular aggregates such as water clusters, surfactant micelles and lipid membranes, are more accurately described by the spatial distribution of their constituent molecules. Therefore, suitable CVs for these systems could be defined using that distribution directly, for example in the form of a density map evaluated on a dense grid over a given volume.

Collective variables based on volumetric maps^63^ were recently introduced in two variants

1. as variables based on a single map, ϕ(**X**) (mapTotal keyword), to bias the number of water molecules in a given region of space; among the applications of mapTotal is, for instance, the dewetting of ion channel pores;^64^
2. as multiple maps combined together, ∑_*i*_ξ_*i*_ϕ_*i*_(**X**), to define a continuous pathway that connects different morphological states of a lipid membrane.

The latter approach, referred to as *Multi-Map*, has been applied to computing the free-energy cost of membrane deformation by embedded transporter proteins,^65^ obtain coarse models of protein conformational changes from density maps,^66^ and to describe the mechanism of curvature generation in cholesterol-rich membranes.^67^

The current implementation of mapTotal and Multi-Map^63^ is specific to NAMD and VMD, because it leverages their implementations of volumetric maps^8,68^ as well as the functionality of the Colvars Dashboard VMD plugin.^7^ Extension to other engines and future improvements will be described elsewhere.

### Alchemical Variable and Lambda-Dynamics

Colvars now implements an alchemical collective variable alchLambda, which is communicated to compatible back-ends–currently, Tinker-HP or NAMD – if their alchemical simulation mode is active.^69^ When the extended-Lagrangian feature is enabled for such a variable, the alchemical simulation becomes driven by Colvars, resulting in a λ-dynamics trajectory. λ-dynamics has existed for several decades^70,71^ but has only been practically accessible to a small fraction of the community so far, thus the Colvars implementation aims at expanding its reach. This feature benefits from improvements in the extended-Lagrangian implementation, especially a new Langevin integrator (BAOA^72^), and reflecting boundary conditions for the extended variable, which is therefore strictly contained within the physically relevant interval [0, 1].

**Figure 4:**
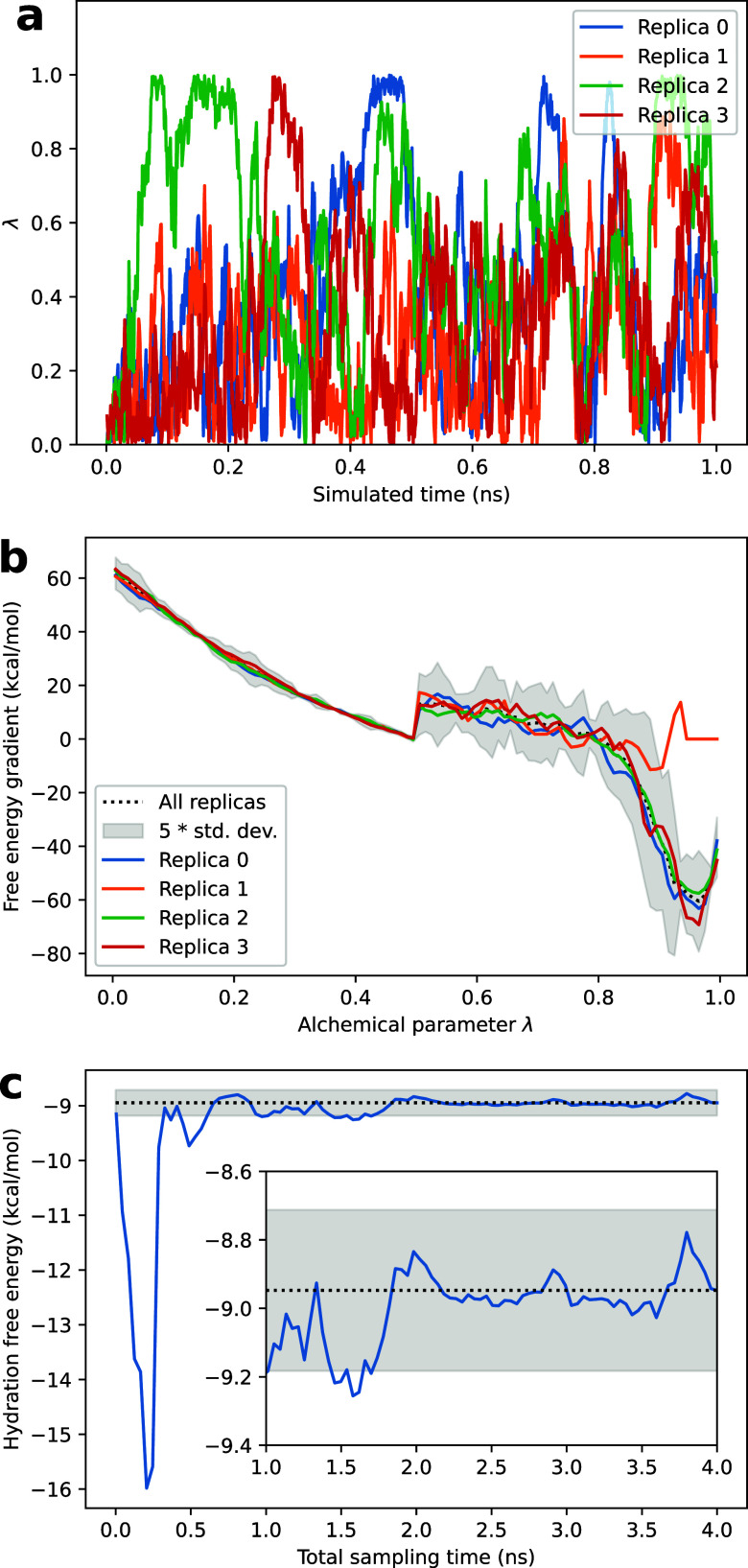
Analysis of an alchemical, multiple-walker lambda-ABF simulation of ketoprofen hydration performed using Colvars and NAMD. (a) Time trajectory of the alchemical parameter λ for the four replicas (“walkers”). (b) Free-energy gradient as a function of the alchemical parameter λ, estimated based on the combined data of four replicas (black) and from each individual replica. The shaded interval is plus or minus the standard deviation between the replicas, weighted by the local sampling of each replica, and multiplied by a factor of 5 for visibility. Replica 1 does not affect the common estimate due to a lack of samples for λ close to 1. The discontinuous change at λ = 0.5 reflects the transition from electrostatic to Lennard-Jones decoupling. (c) Convergence of the estimated free energy of hydration as a function of total sampling time combining the 4 replicas. Inset: vertical zoom on the final segment of the simulation. The shaded area denotes the average plus or minus the error estimate based on the dispersion of the gradient, represented in panel (b).

The alchemical variable can then be used in biasing and free energy calculation methods such as ABF. This combination gives rise to lambda-ABF, a general and simple method for alchemical free energy estimation.^69^ Stochastic diffusion in the alchemical space was found to enhance relaxation in configuration space compared to traditional, fixed-lambda simulations. Lambda-ABF is particularly useful when run over multiple walkers in the mwABF scheme. The alchemical coordinate is another tool in the Colvars toolbox and can be freely combined with other coordinates to create multidimensional biases and other custom methods. As an illustration, the decoupling free energy of the anti-inflammatory drug ketoprofen in TIP3P water was estimated efficiently using short simulations (4 replicas, 1 ns per replica) using Colvars in NAMD. Simulation details can be found in Supporting Information. The results are presented in Figure 4, and discussed in the ABF section below.

The Tinker-HP version of the alchemical variable is compatible with the CPU and GPU implementations. The NAMD implementation is compatible with both the GPU offloading and GPU-resident alchemical simulations^73^ of NAMD 3,^3^ although it does not yet support multiple time-stepping.

## New and Improved Biasing Methods

### Data-Driven Biasing Methods

In addition to the adaptive biasing techniques described in the previous Colvars article,^2^ a range of sophisticated techniques have emerged for integrating experimental measurements and other types of data into simulations. These methods aim to produce structural ensembles consistent with target data while minimizing bias, following the maximum-entropy principle.^74^ They require a forward model to predict target data from the MD ensemble and use a bias potential to align predictions with targets. Here, we describe methods in Colvars based on these concepts, categorizing them into techniques that focus on matching the mean value of an observable and those that target the probability distributions of CVs (which can be viewed as a continuous set of mean values).

#### Methods to Restrain Ensemble Averages

The methods described in this section assume that the experimental data (or other data source) reflects an average of an observable, ξ_*i*_(**X**, **Λ**_***i***_) (forward model), over an ensemble of molecular configurations, **X**, with **Λ**_***i***_ denoting model parameters. To align the simulated mean of ξ_*i*_(**X**, **Λ**_***i***_) with the target value, these methods employ a machine-learning bias potential, *V*_*t*_(ξ_*i*_) (where *t* denotes time), during molecular dynamics simulations. The general expression of *V*_*t*_(ξ_*i*_) follows the principle of maximum entropy:^74^
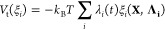
7where the parameter λ_*i*_ controls the mean value of ξ_*i*_(**X**, **Λ**_***i***_). To align the latter mean value with the desired target, the parameter λ_*i*_ is adjusted over time according to a gradient descent optimization:
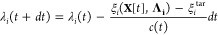
8where ξ_*i*_^tar^ is the target mean value and *c*(*t*) controls the learning rate. The latter rate depends on the observable and can be tailored to ensure efficient convergence.^42,75^

Colvars offers two formulations of these methods: Adaptive Linear Bias (ALB, called alb in Colvars)^75^ and Restrained-Average Dynamics (RAD, called rad in Colvars).^42^ The key distinction is that RAD explicitly accounts for experimental and model uncertainty, thereby reducing the risk of overfitting when handling multiple experimental data sets. In ALB, the target mean value is set precisely to the input experimental value (ξ_*i*_^tar^ = ξ_*i*_^exp^ in eq 8). In contrast, RAD optimizes ξ_*i*_^tar^ to minimize the biasing forces while ensuring that it remains within acceptable error limits. For Gaussian errors the time evolution of ξ_*i*_^tar^ is given by
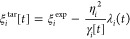
9where η_*i*_ is the estimated experimental error and γ_*i*_ is evolved to set an overall level of agreement between predicted and experimental data (e.g., ∑_*i*_|ξ_*i*_^tar^ – ξ_*i*_^exp^|/*N*η_*i*_ ≈ 1, where *N* is the size of the data set). Inputting both experimental errors and desired agreement levels helps prevent overfitting and excessive bias. Besides uncertainties, RAD explicitly accounts for the model parameters, Λ_*i*_, which, if unknown, can be optimized to reduce bias. However, the current implementation in Colvars only supports observables that are linear functions of the parameters Λ_*i*_, such as the deer CV in the previous section on new coordinates. An example of RAD applied to DEER data is shown in Figure 2. RAD is presently available in a separate branch of Colvars and is in the process of being integrated into the main branch.

#### Methods Targeting CVs Probability Distributions

Probability distributions of molecular observables, such as label distances from DEER or Förster resonance energy transfer (FRET), and pair distances from radiation scattering, are alternative key targets for simulations. Focusing on these derived distributions rather than raw experimental signals (via methods described above) provides a unified framework for integrating these experiments, albeit an indirect one, avoiding the need for experiment-specific formulations.

Probability distributions of CVs can be represented as the ensemble average of density kernel functions *h*_*i*_[ξ(*X*)] (e.g., rectangular or Gaussian), centered on a specific CV value ξ_*i*_, and covering the relevant CV range.^40,76^ Thus, these distributions can be enforced in MD simulations using methods similar to those described above. Roux and co-workers^39,76^ used this formulation to target the spin labels’ distance distributions derived from DEER measurements. In this method, the mean values of the kernel functions are targeted using the restrained-ensemble technique. Namely, *N* simulation replicas are carried out and harmonic potentials, *V*_*i*_, are applied on the mean value of the kernel functions across replicas:
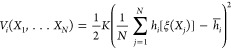
10where *X*_*i*_ denotes the molecular coordinates of replica *i*, *K* is the force constant and *h*_*i*_® is the target mean value. For large *K* and many replicas, the restrained-ensemble technique becomes statistically equivalent to the maximum entropy methods discussed in the previous section.^77^ In practice, Roux et al.’s approach^76^ employs a multiple-copy algorithm with replicas of spin-label moieties only, maintaining a single replica for the protein. Additionally, it applies a mean field approximation to scale the replica average statistics by *N*^2^. The implementation of eq 1 is available in Colvars via the keyword histogramRestraint and is currently applicable to one-dimensional probability distributions.

Besides representing kernel function mean values, probability distributions of CVs are also linked to free energies via logarithmic scaling. This connection has been exploited by Marinelli,^40^ White,^78^ and co-workers to adapt free energy methods, such as metadynamics,^79^ for targeting CVs distributions with an efficient single replica approach. Multiple replicas can be used to speed up convergence as in the multiple-walker metadynamics method,^80^ but are not strictly required. Colvars provides an implementation of this methodology, called ensemble-biased metadynamics (EBMetaD) or ebMeta in Colvars, which can be applied to distributions of arbitrary dimensions. This approach is based on scaling the Gaussian functions in the metadynamics bias potential by the inverse of the target distribution function, referred to as ρ_exp_ (provided in input as a grid function):
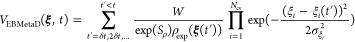
11where *S*_ρ_ represents the target distribution differential entropy (*W* and σ_ξ_*i*__ are Gaussians’ height and width respectively). At convergence, the EBMetaD bias potential is related to the free energy, *A*(**ξ**), via:
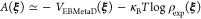
12Namely, EBMetaD is statistically equivalent to the maximum entropy approaches discussed in the previous section when the observables are kernel density functions.^40^ Like the aforementioned methods, EBMetaD has been extended to account for experimental errors^41^ (which are also input as a grid function), using the same formulation as RAD. The extended version of EBMetaD is available in a development version of Colvars and is currently being implemented into the main branch.

### Improvements to the ABF Implementation

The Adaptive Biasing Force method (ABF) is a modified dynamics that enhances the sampling of a low-dimension space of collective variables based on an on-the-fly estimate of the free-energy gradient in that space.^81−83^ Colvars has always included an implementation of ABF.^84^ Since the time of the previous report,^2^ the Colvars/ABF implementation was enriched with the extended-system ABF method (eABF),^85^ which can be coupled to an umbrella-integration free-energy estimator.^86^ The eABF implementation in Colvars has formed the basis for developing methods that combine eABF with other methods such as metadynamics^87,88^ well-tempered metadynamics,^88^ and Gaussian-accelerated MD.^89^ A Poisson integration algorithm has been added for all ABF variants, enabling the code to directly produce integrated free-energy surfaces in dimensions 2 and 3.^90^

Here we also report recent improvements to the multiple-walker, “shared ABF” variant, or mwABF.^15,91^ In shared ABF, different copies of an ABF simulation run in parallel and share their free energy derivative data at finite time intervals, so that they apply nearly the same biasing force, and asymptotically so at long times. In the reworked implementation, each walker keeps a separate copy of its own samples and writes it to a separate set of output files, whereas only the first walker writes the collective free energy and gradient estimates. This allows users to analyze the data from individual walkers, and the dispersion between them can be used as a convergence metric. This is illustrated in Figure 4, describing the alchemical decoupling of ketoprofen from water. Figure 4 displays the time dependence of λ. The λ values for the four replicas, all initialized at 0, cover most of the [0, 1] range. Of note, replica 1 never reaches λ = 1 in this short simulation: this does not affect the reliability of the overall estimate, which combines data from all replicas.

The free energy estimate converges rapidly over time, with fluctuations of 0.1 kcal/mol in the second half of the simulations. The dispersion between the data collected by all the replicas can now easily be analyzed to yield an error estimator.

The free-energy gradient estimated from each replica’s data is now output separately, enabling the dispersion analysis of panel b. As visible in Figure 4a, the replicas’ dynamics in λ space decorrelate rapidly. The benefit of mwABF is that the enhanced diffusion along the CV increases the rate at which replicas become decorrelated in the orthogonal (here, Cartesian) space.^69^ Each replica visits each λ point several times, with a different history. Assuming that this decorrelation has happened, the errors in different replicas are independent, and the error in the common gradient estimate can be computed as the weighted standard deviation between the replicas. The error on the integrated free energy difference is estimated by assuming independence between the errors in different bins.^92^ In this case, the decoupling free energy is estimated as −8.9 ± 0.2 kcal/mol.

The compatibility of mwABF with eABF has been made complete, with improved handling of the data needed by the CZAR free energy estimator^85^ across replicas.

Finally, a more advanced mwABF selection mechanism whereby walkers could be deleted and spawned in regions of lower sampling density has been extended to two-dimensional collective variable spaces. Note that this selection mechanism is only implemented as a NAMD-specific Tcl script, whereas the general mwABF implementation is compatible with LAMMPS, NAMD, Tinker-HP and GROMACS (from 2025 onwards). This is not a major drawback because in our experience, the selection mechanism is not critical in ensuring diversity in the orthogonal space, which is the main benefit of multiple-walker sampling.^69^

### Improvements in Metadynamics-Based Methods

The metadynamics implementation initially detailed in ref (2) included both the original method,^79^ which aims to achieve uniform exploration of CV space, and its well-tempered variant,^93^ which achieves Boltzmann sampling of the free energy in the CV space at a higher, specified temperature. Since then, the metadynamics approach has been extended to target any specified distribution in the CV space via the ensemble-biased metadynamics method,^40,41^ detailed above. Additional improvements to metadynamics have focused on enhancing numerical accuracy and ease of use, which we discuss further here.

#### Boundary Correction for Metadynamics

**Figure 5:**
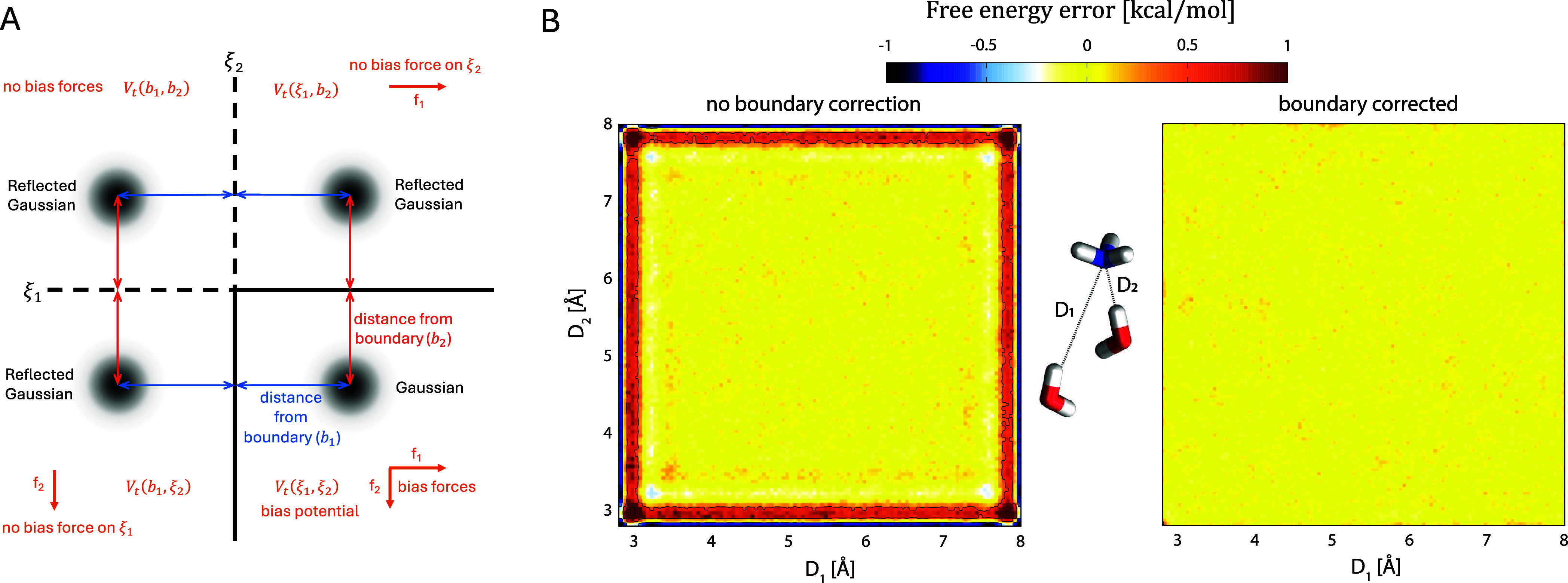
CVs boundary correction for metadynamics and its variants. (A) 2D schematic of the boundary correction method in Colvars. (B) Comparison of metadynamics simulations with (right) and without (left) boundary correction for ammonia coordinated by two water molecules in vacuum. Metadynamics simulations used two CVs (D_1_ and D_2_) representing distances between water oxygen atoms and ammonia nitrogen, with steep confining potentials outside [2.8 Å, 8 Å] and Gaussians added every ps (of height 0.006 kcal/mol and sigma 0.2 Å). The panel compares free energy errors (−*k*_B_*T* ln ρ(*D*_1_, *D*_2_), where ρ is the 2D histogram from the metadynamics sampling) from the last 250 ns of simulation (300 K, 1 fs time step). The boundary correction effectively removes errors from steep walls, as shown in panel B.

Regardless of the metadynamics variant employed, the finite spatial resolution of the Gaussian bias leads to systematic errors near steep walls or mathematical boundaries of CVs. These errors accumulate over the course of the simulation, hindering correct sampling in these regions, which can also cause simulation instabilities during extended runs. As a result, the accuracy of the calculated free energy in these areas can be compromised^94,95^ (Figure 5B; left plot). To address this issue, we implemented a boundary correction in Colvars across arbitrary dimensions, based on concepts from previous research.^94,96,97^ The approach consists in applying reflecting conditions at the boundaries^94,97^ and removing biasing forces components along CVs where exceed their specified boundary limits^96^ (i.e., when the tails of the Gaussians extend beyond themathematical limits of the CVs). This boundary condition can be enabled in Colvars with the keyword useHillsReflection. When activated, additional Gaussians are placed outside the boundaries at locations reflected relative to the original ones and with the same height and width of the original Gaussians (Figure 5A). To improve efficiency, this condition is applied only to Gaussians that are within a cutoff distance from a boundary (by default 6 times the Gaussians sigma, which can be customized with the keyword reflectionRange).

The above treatment also includes Gaussians that are simultaneously reflected across multiple CVs, considering all possible combinations of single, double, or multiple concurrent reflections (Figure 5A). This approach produces a smooth distribution of additional Gaussians on a shell surrounding the exterior of the CV space enclosed by the boundaries. Although the reflected Gaussians are placed outside the boundaries, potentially beyond the mathematically valid region of the CV space, their tails still affect the bias potential and forces within the boundaries. This effect helps reaching stationary conditions and prevents the accumulation of systematic errors. Due to the symmetry of the reflecting condition, biasing force components vanish at the boundaries that are perpendicular to them. Therefore, the aforementioned force components can be safely set to zero outside these boundaries (Figure 5A) if they are not strict mathematical limits (and may be exceeded during the simulation). This avoids discontinuities when crossing boundaries and further reduces systematic errors. Consistently, outside the boundaries we maintain a constant bias potential in the direction of the removed force components, matching the value at the nearest exceeded boundary (Figure 5A).

In summary, the boundary correction method described here is effective and accurate as illustrated here on a model system (Figure 5B). It is compatible with any metadynamics variant in Colvars and requires no additional parameters or significant computational overhead. Additionally, it can be used with both intrinsically limited CVs and those confined by flat-bottom potentials.

When the metadynamics bias is introduced by projecting Gaussians onto a grid (enabled by default), but the boundary correction above is not employed, it is recommended to either:

- extend the grid boundaries to enclose all wall restraints, as detailed in the Colvars documentation, or
- place the grid boundaries at the true mathematical boundaries of each CV whenever possible.

Alongside the above, iIt is also recommended to derive the free energy using statistical reweighting^98−100^ or force-based analysis methods.^101^ These approaches effectively employ free-energy estimators other than the biasing potential itself, and thus may also be useful for assessing the quality of sampling.

#### Usability Improvements in Metadynamics

One of the usability improvements in metadynamics is the ability to specify the width of Gaussian hills along each dimension, in units of the corresponding CVs, using the gaussianSigmas keyword; this option is mutually exclusive with the pre-existing hillWidth option, which specifies the width along all dimensions as a single number of grid points. Lastly, when running multiple-walker metadynamics^80^ it is now possible to use the same Colvars configuration file for all walkers, provided that the MD engine is launched in a multiple-replicas configuration. This is achieved by setting automatically the default value for the replicaID keyword whenever the -multidir option is used in GROMACS, or -partition flag in LAMMPS, the +replicas flag in NAMD, or the replicas keyword in Tinker-HP.

#### On-the-Fly Probability Enhanced Sampling (OPES)

An implementation of On-the-fly Probability Enhanced Sampling (OPES),^102^ a generalization of metadynamics, has been very recently included in Colvars. The implementation is based on the implementation of OPES already distributed with PLUMED,^6^ and therefore retains many of its features save for some changes for the new platform. For example, checkpointing an OPES simulation does not rely on accessory files, but rather on the state file already written by Colvars (NAMD and Tinker-HP) or on the MD engine’s own checkpoint file (GROMACS and LAMMPS).

### Bias-Exchange Schemes

Colvars implicitly supports the temperature-exchange method,^10^ by communicating the value of its current biasing potential to the MD engine, which uses it to compute the probability of exchanges between replicas. Additionally, Colvars provides a direct implementation for other replica-exchange schemes, where the property being exchanged is an umbrella-sampling restraint^103^ or a metadynamics bias.^104^ Both schemes are currently supported using the Colvars scripting interface described below, using scripts available from the NAMD Web site (replica-exchange umbrella sampling) or from the Colvars Web site (bias-exchange metadynamics). As is common with most implementations of replica-exchange schemes, synchronicity and fast communication between replicas are also required. Therefore, this feature is currently limited to NAMD, where script-driven communication between replicas^105^ is available in most typical cluster installations of NAMD.

A noteworthy aspect of the aforementioned implementation of bias-exchange metadynamics is the use of a single configuration file to define the CVs and their metadynamics biases on all replicas simultaneously. During a simulation, only one metadynamics bias is kept active by each replica, with no forces being applied to the CVs that are not involved in that bias. When an exchange is attempted between two replicas, the information accumulated by the two biases is mutually communicated between the respective replicas. If the exchange is successful, the two biases will be swapped and their permutation, will persist until the next exchange attempt and will be restored when continuing a simulation from a checkpoint. This approach has two advantages:

- by performing all exchanges internally within Colvars, all other features of the MD engine remain compatible with the sampling scheme;
- because all CVs are always recorded, regardless of whether forces are applied to them, analyzing the simulated trajectories does not require recomputing from Cartesian coordinates, potentially saving significant storage space.

Lastly, the bias-exchange implementation is easily generalizable to sampling schemes other than the two mentioned above,^103,104^ providing a basis for developing new methods.

### Adiabatic Bias MD

Adiabatic Bias MD (ABMD) is a time-dependent, nonequilibrium biasing method that produces reactive trajectories for rare events by enhancing the forward motion of a progress coordinate ξ_*t*_.^106^ Forward fluctuations of the coordinate are selected by applying a history-dependent harmonic potential *V*_*t*_(ξ_*t*_) centered on the highest value reached by ξ_*t*_ over the past trajectory (high-water mark).
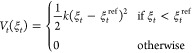
13where ξ_*t*_^ref^ is the high-water mark at time *t*, bounded by a user-defined stopping value ξ^stop^:
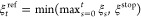
14ABMD (also called ratchet-and-pawl MD) has been successfully applied in particular to protein folding processes.^106−108^ Recently, it has proved efficient at producing diverse binding pathways and poses for a nucleotide ligand inside the cavity of Uncoupling Protein 1, a proton transporter from the mitochondrial inner membrane.^109^

Previously, ABMD had been implemented as a simple scripted bias, showcasing the efficiency of the scripting interface for rapidly implementing new methods. Because the biases work in low-dimension colvar space, the overhead associated with a scripting language is negligible and prototype implementations are efficient enough to be used for production. However, the new, native C++ implementation of ABMD in Colvars makes it more flexible, and portable to all MD back-ends, including GROMACS and LAMMPS.

The C++ implementation adds the abmd bias keyword, with the following options: the name of the collective variable undergoing the bias, the harmonic force constant *k*, the stopping value ξ^stop^, and an optional boolean value indicating that the values should decrease over time rather than increase.

## Custom-Defined Collective Variables and Biases

Extending the functionality of the Colvars library without changing its source code is supported through multiple mechanisms, which are presented here. This section discusses the goal of defining a collective variable ξ(**X**) such as
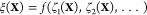
15where ζ_1_(**X**), ζ_2_(**X**),... are functions implemented by Colvars, and *f*(···) is a function defined at runtime by the user. The following sections describe three different approaches to implement *f*(···).

### Linear and Polynomial Superposition

Since its first release, Colvars allows defining the function *f*(···) as a weighted sum of powers of ζ_*i*_(**X**):
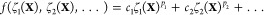
16where *c*_*i*_ are coefficients and *p*_*i*_ integer exponents, defined by the keywords componentCoeff and componentExp, respectively. Typical use cases of this feature range from sums of the same quantity computed over multiple individual molecules (e.g., the total dipole moment), to variables describing multiple states of a whole system.^63^

### Custom Mathematical Functions

In all official releases of NAMD and LAMMPS, as well as patched releases of GROMACS, VMD and Tinker-HP, Colvars allows defining *f*(···) as a closed-form mathematical expression. This functionality is provided by the Lepton library,^61^ originally developed within the OpenMM package;^5^ the same library is also responsible for the automatic calculation of the derivatives of *f*(···) with respect to its arguments, ∂*f*/∂ζ. For example, the residual dipolar coupling (RDC) of a pair of bonded atoms may be defined, save for a multiplicative constant, as
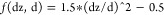
17where the right-hand side of eq 17 is the expression directly usable as the argument of the customFunction keyword and dz and d are, respectively, the labels of a distanceZ and a distance component defined on the pair of atoms.

### Defining Variables and Biases by Scripted Code

In addition to controlling the flow of a simulation (see below), scripting languages may also be used to define additional code at runtime to implement existing collective variables and biases. Two distinct keywords are available:

- scriptedFunction, which defines the root name of two related functions that implement, respectively, *f*(ζ_1_, ζ_2_,...) and its derivatives ∂_ζ_1__*f*(ζ_1_, ζ_2_,...), ∂_ζ_2__*f*(ζ_1_, ζ_2_,...),...; during runtime, ζ_1_, ζ_2_,... are evaluated and their values passed as arguments to the scripted functions;
- scriptedColvarForces, which lets a user-defined function compute and apply biasing forces on multiple CVs, thus allowing implementation of a new type of bias; at runtime, no arguments are provided to this function, which will obtain current values of the CVs and apply forces to them through the Colvars scripting API detailed below.

Currently, this feature supports only the Tcl scripting language, which is available in all installations of NAMD and VMD. Additionally, Colvars in Tinker-HP is also built with an embedded Tcl interpreter that supports callbacks, as used in a recent study.^69^ A typical use case of scripted functions has been the prototyping of path CVs (see New coordinates above) and Euler-angle CVs,^18^ which were later implemented in the library itself. Similarly, Adiabatic Bias MD (ABMD)^106^ was initially implemented as a scripted bias and used in production,^109^ but has since been reimplemented to be available in GROMACS and LAMMPS, which do not support Tcl scripting by default. Support for Python-scripted CVs and biases is under development: its implementation details will be tuned to comply with the packaging systems adopted by each MD engine as their support for Python improves. At the time of writing, among the MD engines supported by Colvars only LAMMPS offers a precompiled package that supports Python callbacks, albeit from a third-party package.

## New or Improved Interfaces

### GROMACS Interface

The Colvars library is now part of the official GROMACS package,^110^ starting with the GROMACS 2024 release. Previous releases (from 2020 to 2023) were also supported through patched versions of the GROMACS code base, or by applying patches manually.

Starting with the 2024 release, a copy of the Colvars library is included in the upstream version of GROMACS and is built by default during the compilation. The library is compatible with most of the GROMACS integrators, GPU offload, thread MPI and the Multiple Time Step feature; starting from version 2025, Colvars can also leverage the multisimulation framework in GROMACS. A few Colvars features such as custom functions and protein secondary structure CVS are not yet included in the standard GROMACS releases, but are available as modified GROMACS releases from the Colvars code repository.

To develop the Colvars-GROMACS interface, the C++ proxy class of Colvars was extended to match the “MDModules” framework of GROMACS, which simplifies adding external modules without modifying core GROMACS files. Other changes, of a more visible nature to the user, were introduced to conform to the GROMACS workflow. For example, all input files (including the GROMACS MD parameters file, the Colvars configuration file and their dependencies) are embedded into the portable binary run input file of GROMACS (*.tpr*) during the ‘*preprocessing*’ step. This ensures the validity of the input files and promotes reproducibility. Furthermore, all Colvars information is stored in the GROMACS checkpoint file (*.cpt*) during the simulation, allowing users to restart or continue a Colvars-GROMACS simulation transparently. Complete documentation is available in the GROMACS manual.^111^

**Figure 6:**
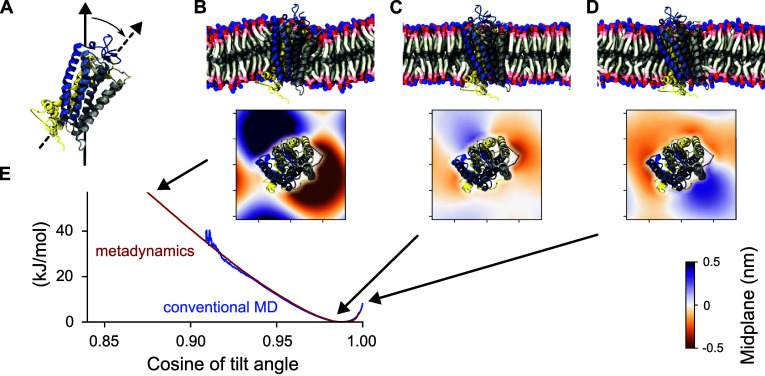
Simulation results, obtained using Colvars and GROMACS, of rhodopsin in a coarse-grained lipid bilayer as a function of the tilt angle θ (A) from the protein’s initial orientation. Shown are simulation snapshots at high tilt (B), at the free-energy minimum (C), and at the initial orientation (D). Each heat map shows the deflection of the bilayer midplane around the protein (in nm) at the corresponding tilt angle. Panel E shows the free energy profile as a function of cos(θ) computed using conventional MD (blue) or metadynamics (red).

Figure 6 illustrates the combined use of Colvars in a typical GROMACS simulation, i.e., a membrane-protein system simulated in coarse-grained representation with the MARTINI force field.^112^ See the Supporting Information for computational details. Lateral diffusion of the protein parallel to the bilayer and rotations around axes perpendicular to it were prevented by restraints applied onto distanceXY and spinAngle variables, respectively. The orientation of the protein during a simulation was quantified by a tilt variable, using as reference the initial coordinates. A 20 μs conventional MD simulation without any biasing forces on the tilt variable shows that the most favorable configuration has a ≈10° angle (cos(θ) ≈ 0.985) with respect to the initial orientation (Figure 6E). Enhanced sampling with metadynamics, also for 20 μs, allows to explore much higher tilt angles (cos(θ) < 0.9). Comparison of the membrane’s midplane deflection maps between the relevant states reveals that the minimum-energy orientation (Figure 6C) minimizes the distortion of the membrane observed in other states (Figure 6B,D).

### Improved LAMMPS and NAMD Interfaces

The Colvars-LAMMPS interface was recently improved by adding support for scripted workflows (see below) through the LAMMPS fix_modify command to send command-line instructions to Colvars. Syntactical differences notwithstanding, this functionality is similar to how Tcl scripting is used in NAMD and VMD. Typical use cases include the ability to customize a run’s input based on the results of prior simulation steps, or controlling when and which output files are written. It is worth clarifying that the latter functionality is available alongside the standard LAMMPS checkpointing mechanism, without replacing it. In fact, using the LAMMPS restart file to checkpoint Colvars has also become more efficient in recent versions, by supporting an unformatted (i.e., binary) representation of the Colvars state data, consistent with other LAMMPS state data such as atomic coordinates, velocities, etc.

The first MD engine that officially supported Colvars is NAMD, owing to the “*GlobalMaster*” infrastructure introduced to support biasing schemes such as steered molecular dynamics, which preceded the introduction of Colvars. The GlobalMaster-based interface between Colvars and NAMD has been extended since its introduction through the following improvements:

- the computation of centers of mass of atom groups and their forces is distributed across parallel tasks;
- when multiple colvar components or biases are defined, their computation can be distributed over multiple threads of the first task;
- the values of volumetric maps^63^ and of the alchemical λ variables^69^ are communicated and processed by Colvars in the same way as atoms and groups centers of mass.

Lastly, although GlobalMaster has traditionally been limited to CPU-based or GPU-offload simulations, it has recently been extended by the NAMD development team to support CPU-based features like Colvars in a GPU-resident run,^3^ an improvement that will be discussed in more detail in a different manuscript.

### Tinker-HP

Colvars is now interfaced with the Tinker-HP package,^113^ which features high-performance implementations of polarizable force fields and machine-learned potentials on CPUs and GPUs. The Colvars library is included in both the CPU and GPU versions of Tinker-HP 1.2.^69^ To build this interface, the C++ proxy class of Colvars was extended with a C layer, which is linked to the Fortran core of Tinker-HP. This provides a template for C and Fortran-based interfaces, further increasing the portability of Colvars. A highlight of the Colvars/Tinker-HP interface is the lambda-dynamics feature (detailed in the section on new coordinates above), which is the core of the lambda-ABF method for alchemical free energy estimation.^69^

Contrary to VMD and NAMD, Tinker-HP does not natively contain a Tcl interpreter. Colvars has been extended with the possibility of building a dedicated, embedded Tcl interpreter. This is enabled in standard Tinker-HP builds, bringing the flexibility of scripted variables and biases implemented as Tcl callbacks. Since Tinker-HP offers no built-in user interface for executing Tcl scripts, we have implemented the Colvars keyword sourceTclFile, which runs a specified script at startup in the Tcl interpreter that is either linked into the back-end or directly embedded in Colvars.

### VMD Interface and the Colvars Dashboard

The Colvars library is linked into executable builds of VMD and can be controlled via a command-line scripting interface based on the Tcl language and the cv command which controls the scripting API detailed below. This interface offers the same functionality to access the molecular topology information available in MD engines. Although no MD integration schemes are available in VMD, the energy and atomic forces produced by Colvars can be analyzed for any configuration loaded in VMD. This feature may be used either to analyze the output of a previous simulation, or to assess the feasibility of adding or modifying a biasing scheme to an ongoing simulation.

In the typical scenario where VMD is used interactively, the above command-line interface provides the basis for a graphical user interface: the Colvars Dashboard.^7^ The Dashboard serves two roles: facilitate the analysis of data sets of molecular configurations to interpret them in terms of colvars, and provide helpers for setting up biased simulations. The configuration of variables and biases can be saved to a file that is directly readable by the supported MD simulation programs (GROMACS, LAMMPS, NAMD, and Tinker-HP).

The interactive display features of the Dashboard include a timeline display to explore the time dependency of the colvars, and pairwise scatterplots to analyze pairwise correlations. Displaying the atoms involved in a variable is useful for troubleshooting atom selections. Colvar gradients with respect to atomic coordinates can be shown as arrows, helping scientists understand the sensitivity of colvars to individual atom coordinates.

New features were added to the Dashboard since the reference publication,^7^ in addition to many small stability and usability improvements. Notably, interactive histograms can now be computed and displayed. Clicking within the histogram changes the displayed frame to that with the closest value of the colvar. Arrow keys navigate the trajectory sorted by colvar values, which is particularly useful for exploring individual modes in multimodal distributions of colvars. The gradient display feature has been extended to all coordinates, including vector ones.

A precomputed colvars trajectory can now be imported from a .colvars.traj file, typically output by a Colvars-enabled simulation. These values are cached and associated with loaded molecular configurations in VMD. This enables the visualization of parameters that cannot be computed in VMD, such as the dynamic alchemical parameter within a lambda-ABF trajectory.^69^ It is also useful for troubleshooting any discrepancy between quantities computed by Colvars in VMD and in MD engines. Lastly, the set of templates for collective variables and biasing algorithms has been extended and improved to cover a much larger set of the Colvars features.

## Code Design and Functionality Improvements

### Scripting Interface: Workflow Control

The range of scientific problems where molecular dynamics (MD) simulations are used and the amount of computational power available to each project are both increasing steadily. Therefore, many simulation packages also offer the ability to customize or automate the flow of an MD simulation via scripting languages. The two languages most often used in MD simulations, Tcl and Python, support this goal in distinct ways, such as allowing easy integration with simulation software (Tcl) or leveraging a rich set of features and knowledge base (Python). Furthermore, certain MD simulation packages, such as LAMMPS, provide control instructions that provide, in essence, a specialized scripting language for that application. Colvars supports each of the three above languages: currently, the most mature and feature-rich interface is with the Tcl language (specifically, via the cv command), available in all deployments of NAMD and VMD, where it also forms the basis of the Colvars Dashboard graphical user interface described previously.

Through scripted code, individual collective variables and their biases may be added, removed, or modified midway through a simulation, which allows for fine-tuning performance or dynamically redefining the enhanced sampling scheme. Notable examples of this feature are the bias-exchange metadynamics method (see the section on metadynamics improvements above), or the implementation of path-learning methods such as milestoning using the ScMiles2 software,^114^ both of which are implemented in Tcl.

### Continuous Integration (CI) Testing

Integration and testing of all new code are performed via the public GitHub repository https://github.com/Colvars/colvars. To ensure the reliability of the library in any new release of each supported package, the Colvars library receives continuous updates and improvements and is deployed following a rolling-release model. To support this model, integration of any source code revisions is performed conditionally on the outcome of automated tests of the stand-alone library and the back-end engines. This measure guarantees that changes maintain the correctness of the computations across all supported packages and platforms.

## Conclusions

Colvars has been enhanced in many respects over the past decade, making it more broadly available and more useful to the community. Nonetheless, progress is not as immediately reflected in the numbering scheme of its versions: Colvars does not have major releases, because it is made available as part of the standard distribution of mainstream molecular simulation and analysis packages. Beyond that distribution method, advanced users may obtain the Colvars source code directly from its repository, accessing a continuous stream of well-tested new features and improvements. Experimental features are also available in development branches, as they are discussed with the associated issues or pull requests, following modern software development practices. This manuscript surveyed improvements made since the Colvars reference paper was published,^2^ with particular emphasis on novel features and increased portability. Enhancements in performance and scalability, including those currently under development, will be covered in separate publications.

In their early days, MD simulations were primarily used to illustrate models previously set forth by experimental measurements. However, dramatic improvement in the performance of MD simulations now allows for prediction of the outcome of experiments yet to be performed. Therefore, MD is especially effective at addressing problems that are otherwise challenging for methods such as experimental structure detection and machine-learning models trained on those structures. The applications shown here demonstrate, for example, that collective variable-based methods are effective at estimating the strength of binding between proteins and their ligands, or the mechanism of interaction between proteins and their surrounding lipid membrane. Importantly, by close integration of, and codistribution with, Colvars and the packages it supports, we minimize the barriers to access such enhanced-sampling approaches, allowing widespread adoption of state-of-the-art methodologies to address critical challenges remaining in molecular science.

## Supporting Information Available

Computational details for simulations and mathematical expression for biasing forces in a moving frame of reference (PDF)

## References

[ref1] Hénin J.; Lelièvre T.; Shirts M. R.; Valsson O.; Delemotte L. Enhanced Sampling Methods for Molecular Dynamics Simulations [Article v1.0]. Living J. Comput. Mol. Sci. 2022, 4, 1583 10.33011/livecoms.4.1.1583.

[ref2] Fiorin G.; Klein M. L.; Hénin J. Using collective variables to drive molecular dynamics simulations. Mol. Phys. 2013, 111, 3345–3362. 10.1080/00268976.2013.813594.

[ref3] Phillips J.; Hardy D.; Maia J.; Stone J.; Ribeiro J.; Bernardi R.; Buch R.; Fiorin G.; Hénin J.; Jiang W.; et al. Scalable molecular dynamics on CPU and GPU architectures with NAMD. J. Chem. Phys. 2020, 153, 044130 10.1063/5.0014475. 32752662 PMC7395834

[ref4] Páll S.; Zhmurov A.; Bauer P.; Abraham M.; Lundborg M.; Gray A.; Hess B.; Lindahl E. Heterogeneous parallelization and acceleration of molecular dynamics simulations in GROMACS. J. Chem. Phys. 2020, 153, 134110 10.1063/5.0018516. 33032406

[ref5] Eastman P.; Galvelis R.; Peláez R. P.; Abreu C. R. A.; Farr S. E.; Gallicchio E.; Gorenko A.; Henry M. M.; Hu F.; Huang J.; et al. OpenMM 8: Molecular Dynamics Simulation with Machine Learning Potentials. J. Phys. Chem. B 2024, 128, 109–116. 10.1021/acs.jpcb.3c06662. 38154096 PMC10846090

[ref6] Tribello G. A.; Bonomi M.; Branduardi D.; Camilloni C.; Bussi G. PLUMED 2: New feathers for an old bird. Comput. Phys. Commun. 2014, 185, 604–613. 10.1016/j.cpc.2013.09.018.

[ref7] Hénin J.; Lopes L. J. S.; Fiorin G. Human Learning for Molecular Simulations: The Collective Variables Dashboard in VMD. J. Chem. Theory Comput. 2022, 18, 1945–1956. 10.1021/acs.jctc.1c01081. 35143194

[ref8] Humphrey W.; Dalke A.; Schulten K. VMD: visual molecular dynamics. J. Mol. Graph. 1996, 14, 33–38. 10.1016/0263-7855(96)00018-5. 8744570

[ref9] Thompson A. P.; Aktulga H. M.; Berger R.; Bolintineanu D. S.; Brown W. M.; Crozier P. S.; in’t Veld P. J.; Kohlmeyer A.; Moore S. G.; Nguyen T. D.; et al. LAMMPS - a flexible simulation tool for particle-based materials modeling at the atomic, meso, and continuum scales. Comput. Phys. Commun. 2022, 271, 108171 10.1016/j.cpc.2021.108171.

[ref10] Sugita Y.; Okamoto Y. Replica-exchange molecular dynamics method for protein folding. Chem. Phys. Lett. 1999, 314, 141–151. 10.1016/S0009-2614(99)01123-9.

[ref11] Teo I.; Mayne C. G.; Schulten K.; Lelièvre T. Adaptive Multilevel Splitting Method for Molecular Dynamics Calculation of Benzamidine-Trypsin Dissociation Time. J. Chem. Theory Comput. 2016, 12, 2983–2989. 10.1021/acs.jctc.6b00277. 27159059 PMC5724379

[ref12] Lopes L. J. S.; Lelièvre T. Analysis of the adaptive multilevel splitting method on the isomerization of alanine dipeptide. J. Comput. Chem. 2019, 40, 1198–1208. 10.1002/jcc.25778. 30697777

[ref13] Elber R. Milestoning: An efficient approach for atomically detailed simulations of kinetics in biophysics. Annual review of biophysics 2020, 49, 69–85. 10.1146/annurev-biophys-121219-081528. 32375019

[ref14] Zuckerman D. M.; Chong L. T. Weighted ensemble simulation: review of methodology, applications, and software. Annual review of biophysics 2017, 46, 43–57. 10.1146/annurev-biophys-070816-033834. PMC5896317 28301772

[ref15] Comer J.; Phillips J. C.; Schulten K.; Chipot C. Multiple-replica strategies for free-energy calculations in NAMD: multiple-walker adaptive biasing force and walker selection rules. J. Chem. Theory Comput. 2014, 10, 5276–5285. 10.1021/ct500874p. 26583211

[ref16] Woo H.-J.; Roux B. Calculation of absolute protein–ligand binding free energy from computer simulations. Proc. Natl. Acad. Sci. U.S.A. 2005, 102, 6825–6830. 10.1073/pnas.0409005102. 15867154 PMC1100764

[ref17] Gumbart J. C.; Roux B.; Chipot C. Standard Binding Free Energies from Computer Simulations: What Is the Best Strategy?. J. Chem. Theory Comput. 2013, 9, 794–802. 10.1021/ct3008099. 23794960 PMC3685508

[ref18] Fu H.; Cai W.; Hénin J.; Roux B.; Chipot C. New Coarse Variables for the Accurate Determination of Standard Binding Free Energies. J. Chem. Theory Comput. 2017, 13, 5173–5178. 10.1021/acs.jctc.7b00791. 28965398

[ref19] Coutsias E. A.; Seok C.; Dill K. A. Using quaternions to calculate RMSD. J. Comput. Chem. 2004, 25, 1849–1857. 10.1002/jcc.20110. 15376254

[ref20] Fu H.; Gumbart J. C.; Chen H.; Shao X.; Cai W.; Chipot C. BFEE: A User-Friendly Graphical Interface Facilitating Absolute Binding Free-Energy Calculations. J. Chem. Inf. Model. 2018, 58, 556–560. 10.1021/acs.jcim.7b00695. 29405709 PMC5869121

[ref21] Fu H.; Chen H.; Cai W.; Shao X.; Chipot C. BFEE2: Automated, Streamlined, and Accurate Absolute Binding Free-Energy Calculations. J. Chem. Inf. Model. 2021, 61, 2116–2123. 10.1021/acs.jcim.1c00269. 33906354

[ref22] De Donder T. L’affinit é; Gauthier–Villars: Paris, 1927.

[ref23] Kirkwood J. G. Statistical mechanics of fluid mixtures. J. Chem. Phys. 1935, 3, 300–313. 10.1063/1.1749657.

[ref24] E W.; Ren W.; Vanden-Eijnden E. String method for the study of rare events. Phys. Rev. B 2002, 66, 052301 10.1103/PhysRevB.66.052301. 16851751

[ref25] Pan A. C.; Sezer D.; Roux B. Finding transition pathways using the string method with swarms of trajectories. J. Phys. Chem. B 2008, 112, 3432–3440. 10.1021/jp0777059. 18290641 PMC2757167

[ref26] Chen H.; Ogden D.; Pant S.; Roux B.; Moradi M.; Cai W.; Tajkhorshid E.; Chipot C. A companion guide to the string method with swarms of trajectories. Characterization, performance, and pitfalls. J. Chem. Theory Comput. 2022, 18, 1406–1422. 10.1021/acs.jctc.1c01049. 35138832 PMC8904302

[ref27] Jung H.; Covino R.; Arjun A.; Leitold C.; Dellago C.; Bolhuis P. G.; Hummer G. Machine-guided path sampling to discover mechanisms of molecular self-organization. Nature Comput. Sci. 2023, 3, 334–345. 10.1038/s43588-023-00428-z. 38177937 PMC10766509

[ref28] Chen H.; Roux B.; Chipot C. Discovering reaction pathways, slow variables, and committor probabilities with machine learning. J. Chem. Theory Comput. 2023, 19, 4414–4426. 10.1021/acs.jctc.3c00028. 37224455 PMC11372462

[ref29] Kang P.; Trizio E.; Parrinello M. Computing the committor with the committor to study the transition state ensemble. Nature Comput. Sci. 2024, 4, 451–460. 10.1038/s43588-024-00645-0. 38839932

[ref30] Peters B. Reaction coordinates and mechanistic hypothesis tests. Annu. Rev. Phys. Chem. 2016, 67, 669–690. 10.1146/annurev-physchem-040215-112215. 27090846

[ref31] Rogal J. Reaction coordinates in complex systems—A perspective. Eur. Phys. J. B 2021, 94, 223 10.1140/epjb/s10051-021-00233-5.

[ref32] Chipot C. Free energy methods for the description of molecular processes. Annu. Rev. Biophys. 2023, 52, 113–138. 10.1146/annurev-biophys-062722-093258. 36626763

[ref33] Branduardi D.; Gervasio F. L.; Parrinello M. From A to B in free energy space. J. Chem. Phys. 2007, 126, 054103 10.1063/1.2432340. 17302470

[ref34] Díaz Leines G.; Ensing B. Path finding on high-dimensional free energy landscapes. Phys. Rev. Lett. 2012, 109, 020601 10.1103/PhysRevLett.109.020601. 23030145

[ref35] Fu H.; Chen H.; Blazhynska M.; Coderc Goulard; de Lacam E.; Szczepaniak F.; Pavlova A.; Shao X.; Gumbart J. C.; Dehez F.; Roux B.; et al. Accurate determination of protein:ligand standard binding free energies from molecular dynamics simulations. Nat. Protoc. 2022, 17, 1114–1141. 10.1038/s41596-021-00676-1. 35277695 PMC10082674

[ref36] Salari R.; Joseph T.; Lohia R.; Hénin J.; Brannigan G. A Streamlined, General Approach for Computing Ligand Binding Free Energies and Its Application to GPCR-Bound Cholesterol. J. Chem. Theory Comput. 2018, 14, 6560–6573. 10.1021/acs.jctc.8b00447. 30358394 PMC6467757

[ref37] Santiago-McRae E.; Ebrahimi M.; Sandberg J. W.; Brannigan G.; Hénin J. Computing absolute binding affinities by Streamlined Alchemical Free Energy Perturbation [Article v1.0]. Living J. Comput. Mol. Sci. 2023, 5, 2067 10.33011/livecoms.5.1.2067.

[ref38] Ebrahimi M.; Hénin J. Symmetry-Adapted Restraints for Binding Free Energy Calculations. J. Chem. Theory Comput. 2022, 18, 2494–2502. 10.1021/acs.jctc.1c01235. 35230113

[ref39] Islam S. M.; Stein R. A.; Mchaourab H. S.; Roux B. Structural Refinement from Restrained-Ensemble Simulations Based on EPR/DEER Data: Application to T4 Lysozyme. J. Phys. Chem. B 2013, 117, 4740–4754. 10.1021/jp311723a. 23510103 PMC3684008

[ref40] Marinelli F.; Faraldo-Gómez J. Ensemble-Biased Metadynamics: A Molecular Simulation Method to Sample Experimental Distributions. Biophys. J. 2015, 108, 2779–2782. 10.1016/j.bpj.2015.05.024. 26083917 PMC4472218

[ref41] Hustedt E. J.; Marinelli F.; Stein R. A.; Faraldo-Gómez J. D.; Mchaourab H. S. Confidence Analysis of DEER Data and Its Structural Interpretation with Ensemble-Biased Metadynamics. Biophys. J. 2018, 115, 1200–1216. 10.1016/j.bpj.2018.08.008. 30197182 PMC6170522

[ref42] Marinelli F.; Fiorin G. Structural Characterization of Biomolecules through Atomistic Simulations Guided by DEER Measurements. Structure 2019, 27, 359–370.e12. 10.1016/j.str.2018.10.013. 30528595 PMC6860373

[ref43] Schmidt T.; Wälti M. A.; Baber J. L.; Hustedt E. J.; Clore G. M. Long Distance Measurements up to 160 Å in the GroEL Tetradecamer Using Q-Band DEER EPR Spectroscopy. Angew. Chem., Int. Ed. 2016, 55, 15905–15909. 10.1002/anie.201609617. PMC5166617 27860003

[ref44] Milov A.; Ponomarev A.; Tsvetkov Y. Electron-electron double resonance in electron spin echo: Model biradical systems and the sensitized photolysis of decalin. Chem. Phys. Lett. 1984, 110, 67–72. 10.1016/0009-2614(84)80148-7.

[ref45] Milov A. D.; Tsvetkov Y. D. Double electron-electron resonance in electron spin echo: Conformations of spin-labeled poly-4-vinilpyridine in glassy solutions. Appl. Magn. Reson. 1997, 12, 495–504. 10.1007/BF03164129.

[ref46] Jeschke G.; Chechik V.; Ionita P.; Godt A.; Zimmermann H.; Banham J.; Timmel C. R.; Hilger D.; Jung H. DeerAnalysis2006—a comprehensive software package for analyzing pulsed ELDOR data. Appl. Magn. Reson. 2006, 30, 473–498. 10.1007/BF03166213.

[ref47] Brandon S.; Beth A. H.; Hustedt E. J. The global analysis of DEER data. J. Magn. Reson. 2012, 218, 93–104. 10.1016/j.jmr.2012.03.006. 22578560 PMC3608411

[ref48] Edwards T. H.; Stoll S. A Bayesian approach to quantifying uncertainty from experimental noise in DEER spectroscopy. J. Magn. Reson. 2016, 270, 87–97. 10.1016/j.jmr.2016.06.021. 27414762 PMC4996738

[ref49] Hornik K. Approximation capabilities of multilayer feedforward networks. Neural Networks 1991, 4, 251–257. 10.1016/0893-6080(91)90009-T.

[ref50] Mardt A.; Pasquali L.; Wu H.; Noé F. VAMPnets for deep learning of molecular kinetics. Nat. Commun. 2018, 9, 5 10.1038/s41467-017-02388-1. 29295994 PMC5750224

[ref51] Wang Y.; Ribeiro J. M. L.; Tiwary P. Past–future information bottleneck for sampling molecular reaction coordinate simultaneously with thermodynamics and kinetics. Nat. Commun. 2019, 10, 3573 10.1038/s41467-019-11405-4. 31395868 PMC6687748

[ref52] Chen W.; Sidky H.; Ferguson A. L. Nonlinear discovery of slow molecular modes using state-free reversible VAMPnets. J. Chem. Phys. 2019, 150, 214114 10.1063/1.5092521. 31176319

[ref53] Sidky H.; Chen W.; Ferguson A. L. Machine learning for collective variable discovery and enhanced sampling in biomolecular simulation. Mol. Phys. 2020, 118, e1737742 10.1080/00268976.2020.1737742.

[ref54] Zhang J.; Lei Y.-K.; Zhang Z.; Chang J.; Li M.; Han X.; Yang L.; Yang Y. I.; Gao Y. Q. A Perspective on Deep Learning for Molecular Modeling and Simulations. J. Phys. Chem. A 2020, 124, 6745–6763. 10.1021/acs.jpca.0c04473. 32786668

[ref55] Bonati L.; Trizio E.; Rizzi A.; Parrinello M. A unified framework for machine learning collective variables for enhanced sampling simulations: mlcolvar. J. Chem. Phys. 2023, 159, 014801 10.1063/5.0156343. 37409767

[ref56] Ketkaew R.; Luber S. DeepCV: A Deep Learning Framework for Blind Search of Collective Variables in Expanded Configurational Space. J. Chem. Inf. Model. 2022, 62, 6352–6364. 10.1021/acs.jcim.2c00883. 36445176

[ref57] Ramil M.; Boudier C.; Goryaeva A. M.; Marinica M.-C.; Maillet J.-B. On Sampling Minimum Energy Path. J. Chem. Theory Comput. 2022, 18, 5864–5875. 10.1021/acs.jctc.2c00314. 36073162

[ref58] Chen H.; Liu H.; Feng H.; Fu H.; Cai W.; Shao X.; Chipot C. MLCV: Bridging Machine-Learning-Based Dimensionality Reduction and Free-Energy Calculation. J. Chem. Inf. Model. 2022, 62, 1–8. 10.1021/acs.jcim.1c01010. 34939790

[ref59] Kleiman D. E.; Nadeem H.; Shukla D. Adaptive Sampling Methods for Molecular Dynamics in the Era of Machine Learning. J. Phys. Chem. B 2023, 127, 10669–10681. 10.1021/acs.jpcb.3c04843. 38081185

[ref60] Fu H.; Bian H.; Shao X.; Cai W. Collective Variable-Based Enhanced Sampling: From Human Learning to Machine Learning. J. Phys. Chem. Lett. 2024, 15, 1774–1783. 10.1021/acs.jpclett.3c03542. 38329095

[ref61] Eastman P.; Pande V. In GPU Computing Gems Jade ed.; Hwu W.-M. W., Ed.; Applications of GPU Computing Series; Morgan Kaufmann: Boston, 2012; pp 399–407.

[ref62] Fu H.; Liu H.; Xing J.; Zhao T.; Shao X.; Cai W. Deep-Learning-Assisted Enhanced Sampling for Exploring Molecular Conformational Changes. J. Phys. Chem. B 2023, 127, 9926–9935. 10.1021/acs.jpcb.3c05284. 37947397

[ref63] Fiorin G.; Marinelli F.; Faraldo-Gómez J. D. Direct Derivation of Free Energies of Membrane Deformation and Other Solvent Density Variations From Enhanced Sampling Molecular Dynamics. J. Comput. Chem. 2020, 41, 449–459. 10.1002/jcc.26075. 31602694 PMC8388148

[ref64] Coronel L.; Muccio G. D.; Rothberg B.; Giacomello A.; Carnevale V. Lipid-mediated hydrophobic gating in the BK potassium channel. 2024; https://arxiv.org/abs/2405.04644. 10.1038/s41467-025-61638-9 PMC12335557 40783487

[ref65] Zhou W.; Fiorin G.; Anselmi C.; Karimi-Varzaneh H. A.; Poblete H.; Forrest L. R.; Faraldo-Gómez J. D. Large-scale state-dependent membrane remodeling by a transporter protein. eLife 2019, 8, e50576 10.7554/eLife.50576. 31855177 PMC6957315

[ref66] Vant J. W.; Sarkar D.; Streitwieser E.; Fiorin G.; Skeel R.; Vermaas J. V.; Singharoy A. Data-guided Multi-Map variables for ensemble refinement of molecular movies. J. Chem. Phys. 2020, 153, DOI: 10.1063/5.0022433. PMC7714525 33291927

[ref67] Fiorin G.; Forrest L. R.; Faraldo-Gómez J. D. Membrane free-energy landscapes derived from atomistic dynamics explain nonuniversal cholesterol-induced stiffening. PNAS Nexus 2023, 2, pgad269 10.1093/pnasnexus/pgad269. 37637198 PMC10456217

[ref68] Wells D. B.; Abramkina V.; Aksimentiev A. Exploring transmembrane transport through α-hemolysin with grid-steered molecular dynamics. J. Chem. Phys. 2007, 127, 125101 10.1063/1.2770738. 17902937 PMC2888542

[ref69] Lagardère L.; Maurin L.; Adjoua O.; El Hage K.; Monmarché P.; Piquemal J.-P.; Hénin J. Lambda-ABF: Simplified, Portable, Accurate, and Cost-Effective Alchemical Free-Energy Computation. J. Chem. Theory Comput. 2024, 20, 4481–4498. 10.1021/acs.jctc.3c01249. 38805379

[ref70] Tidor B. Simulated annealing on free energy surfaces by a combined molecular dynamics and Monte Carlo approach. J. Phys. Chem. 1993, 97, 1069–1073. 10.1021/j100107a015.

[ref71] Kong X.; Brooks C. L. III λ-dynamics: A new approach to free energy calculations. J. Chem. Phys. 1996, 105, 2414–2423. 10.1063/1.472109.

[ref72] Bou-Rabee N.; Owhadi H. Long-Run Accuracy of Variational Integrators in the Stochastic Context. SIAM Journal on Numerical Analysis 2010, 48, 278–297. 10.1137/090758842.

[ref73] Chen H.; Maia J. D. C.; Radak B. K.; Hardy D. J.; Cai W.; Chipot C.; Tajkhorshid E. Boosting Free-Energy Perturbation Calculations with GPU-Accelerated NAMD. J. Chem. Inf. Model. 2020, 60, 5301–5307. 10.1021/acs.jcim.0c00745. 32805108 PMC7686227

[ref74] Pitera J. W.; Chodera J. D. On the Use of Experimental Observations to Bias Simulated Ensembles. J. Chem. Theory Comput. 2012, 8, 3445–3451. 10.1021/ct300112v. 26592995

[ref75] White A. D.; Voth G. A. Efficient and Minimal Method to Bias Molecular Simulations with Experimental Data. J. Chem. Theory Comput. 2014, 10, 3023–3030. 10.1021/ct500320c. 26588273

[ref76] Roux B.; Islam S. M. Restrained-Ensemble Molecular Dynamics Simulations Based on Distance Histograms from Double Electron–Electron Resonance Spectroscopy. J. Phys. Chem. B 2013, 117, 4733–4739. 10.1021/jp3110369. 23510121 PMC3683991

[ref77] Roux B.; Weare J. On the statistical equivalence of restrained-ensemble simulations with the maximum entropy method. J. Chem. Phys. 2013, 138, 084107 10.1063/1.4792208. 23464140 PMC3598863

[ref78] White A. D.; Dama J. F.; Voth G. A. Designing Free Energy Surfaces That Match Experimental Data with Metadynamics. J. Chem. Theory Comput. 2015, 11, 2451–2460. 10.1021/acs.jctc.5b00178. 26575545

[ref79] Laio A.; Parrinello M. Escaping free energy minima. Proc. Natl. Acad. Sci. U.S.A. 2002, 99, 12562–12565. 10.1073/pnas.202427399. 12271136 PMC130499

[ref80] Raiteri P.; Laio A.; Gervasio F. L.; Micheletti C.; Parrinello M. Efficient Reconstruction of Complex Free Energy Landscapes by Multiple Walkers Metadynamics. J. Phys. Chem. B 2006, 110, 3533–3539. 10.1021/jp054359r. 16494409

[ref81] Darve E.; Pohorille A. Calculating free energies using average force. J. Chem. Phys. 2001, 115, 9169–9183. 10.1063/1.1410978.

[ref82] Hénin J.; Chipot C. Overcoming free energy barriers using unconstrained molecular dynamics simulations. J. Chem. Phys. 2004, 121, 2904–2914. 10.1063/1.1773132. 15291601

[ref83] Darve E.; Rodríguez-Gómez D.; Pohorille A. Adaptive biasing force method for scalar and vector free energy calculations. J. Chem. Phys. 2008, 128, 144120 10.1063/1.2829861. 18412436

[ref84] Hénin J.; Fiorin G.; Chipot C.; Klein M. L. Exploring multidimensional free energy landscapes using time-dependent biases on collective variables. J. Chem. Theory Comput. 2010, 6, 35–47. 10.1021/ct9004432. 26614317 PMC12908704

[ref85] Lesage A.; Lelièvre T.; Stoltz G.; Hénin J. Smoothed Biasing Forces Yield Unbiased Free Energies with the Extended-System Adaptive Biasing Force Method. J. Phys. Chem. B 2017, 121, 3676–3685. 10.1021/acs.jpcb.6b10055. 27959559 PMC5402294

[ref86] Fu H.; Shao X.; Chipot C.; Cai W. Extended Adaptive Biasing Force algorithm. An on-the-fly implementation for accurate free-energy calculations. J. Chem. Theory Comput. 2016, 12, 3506–3513. 10.1021/acs.jctc.6b00447. 27398726

[ref87] Fu H.; Zhang H.; Chen H.; Shao X.; Chipot C.; Cai W. Zooming across the free-energy landscape: shaving barriers, and flooding valleys. J. Phys. Chem. Lett. 2018, 9, 4738–4745. 10.1021/acs.jpclett.8b01994. 30074802

[ref88] Fu H.; Shao X.; Cai W.; Chipot C. Taming Rugged Free Energy Landscapes Using an Average Force. Acc. Chem. Res. 2019, 52, 3254–3264. 10.1021/acs.accounts.9b00473. 31680510

[ref89] Chen H.; Fu H.; Chipot C.; Shao X.; Cai W. Overcoming Free-Energy Barriers with a Seamless Combination of a Biasing Force and a Collective Variable-Independent Boost Potential. J. Chem. Theory Comput. 2021, 17, 3886–3894. 10.1021/acs.jctc.1c00103. 34106706

[ref90] Hénin J. Fast and Accurate Multidimensional Free Energy Integration. J. Chem. Theory Comput. 2021, 17, 6789–6798. 10.1021/acs.jctc.1c00593. 34665624

[ref91] Minoukadeh K.; Chipot C.; Lelièvre T. Potential of Mean Force Calculations: A Multiple-Walker Adaptive Biasing Force Approach. J. Chem. Theory Comput. 2010, 6, 1008–1017. 10.1021/ct900524t.

[ref92] Comer J.; Gumbart J. C.; Hénin J.; Lelièvre T.; Pohorille A.; Chipot C. The adaptive biasing force method: everything you always wanted to know but were afraid to ask. J. Phys. Chem. B 2015, 119, 1129–1151. 10.1021/jp506633n. 25247823 PMC4306294

[ref93] Barducci A.; Bussi G.; Parrinello M. Well-tempered metadynamics: a smoothly converging and tunable free-energy method. Phys. Rev. Lett. 2008, 100, 020603 10.1103/PhysRevLett.100.020603. 18232845

[ref94] Crespo Y.; Marinelli F.; Pietrucci F.; Laio A. Metadynamics convergence law in a multidimensional system. Phys. Rev. E 2010, 81, 055701 10.1103/PhysRevE.81.055701. 20866290

[ref95] McGovern M.; de Pablo J. A boundary correction algorithm for metadynamics in multiple dimensions. J. Chem. Phys. 2013, 139, 084102 10.1063/1.4818153. 24006969

[ref96] Baftizadeh F.; Cossio P.; Pietrucci F.; Laio A. Protein Folding and Ligand-Enzyme Binding from Bias-Exchange Metadynamics Simulations. Curr. Phys. Chem. 2012, 2, 79–91. 10.2174/1877946811202010079.

[ref97] Bussi G.; Laio A.; Parrinello M. Equilibrium Free Energies from Nonequilibrium Metadynamics. Phys. Rev. Lett. 2006, 96, 090601 10.1103/PhysRevLett.96.090601. 16606249

[ref98] Marinelli F.; Pietrucci F.; Laio A.; Piana S. A Kinetic Model of Trp-Cage Folding from Multiple Biased Molecular Dynamics Simulations. PLOS Computational Biology 2009, 5, 1–18. 10.1371/journal.pcbi.1000452. PMC2711228 19662155

[ref99] Biarnés X.; Pietrucci F.; Marinelli F.; Laio A. METAGUI. A VMD interface for analyzing metadynamics and molecular dynamics simulations. Comput. Phys. Commun. 2012, 183, 203–211. 10.1016/j.cpc.2011.08.020.

[ref100] Giorgino T.; Laio A.; Rodriguez A. METAGUI 3: A graphical user interface for choosing the collective variables in molecular dynamics simulations. Comput. Phys. Commun. 2017, 217, 204–209. 10.1016/j.cpc.2017.04.009.

[ref101] Marinelli F.; Faraldo-Gómez J. D. Force-Correction Analysis Method for Derivation of Multidimensional Free-Energy Landscapes from Adaptively Biased Replica Simulations. J. Chem. Theory Comput. 2021, 17, 6775–6788. 10.1021/acs.jctc.1c00586. 34669402

[ref102] Invernizzi M.; Parrinello M. Rethinking Metadynamics: From Bias Potentials to Probability Distributions. J. Phys. Chem. Lett. 2020, 11, 2731–2736. 10.1021/acs.jpclett.0c00497. 32191470

[ref103] Fukunishi H.; Watanabe O.; Takada S. On the Hamiltonian replica exchange method for efficient sampling of biomolecular systems: Application to protein structure prediction. J. Chem. Phys. 2002, 116, 9058–9067. 10.1063/1.1472510.

[ref104] Piana S.; Laio A. A bias-exchange approach to protein folding. J. Phys. Chem. B 2007, 111, 4553–4559. 10.1021/jp067873l. 17419610

[ref105] Jiang W.; Phillips J. C.; Huang L.; Fajer M.; Meng Y.; Gumbart J. C.; Luo Y.; Schulten K.; Roux B. Generalized scalable multiple copy algorithms for molecular dynamics simulations in NAMD. Comput. Phys. Commun. 2014, 185, 908–916. 10.1016/j.cpc.2013.12.014. 24944348 PMC4059768

[ref106] Marchi M.; Ballone P. Adiabatic bias molecular dynamics: A method to navigate the conformational space of complex molecular systems. J. Chem. Phys. 1999, 110, 3697–3702. 10.1063/1.478259.

[ref107] Paci E.; Karplus M. Forced unfolding of fibronectin type 3 modules: an analysis by biased molecular dynamics simulations. J. Mol. Biol. 1999, 288, 441–459. 10.1006/jmbi.1999.2670. 10329153

[ref108] Beccara S. A.; Škrbić T.; Covino R.; Faccioli P. Dominant folding pathways of a WW domain. Proc. Natl. Acad. Sci. U. S. A. 2012, 109, 2330–2335. 10.1073/pnas.1111796109. 22308345 PMC3289289

[ref109] Gagelin A.; Largeau C.; Masscheleyn S.; Piel M. S.; Calderón-Mora D.; Bouillaud F.; Hénin J.; Miroux B. Molecular determinants of inhibition of UCP1-mediated respiratory uncoupling. Nat. Commun. 2023, 14, 2594 10.1038/s41467-023-38219-9. 37147287 PMC10162991

[ref110] Abraham M. J.; Murtola T.; Schulz R.; Páll S.; Smith J. C.; Hess B.; Lindahl E. GROMACS: High performance molecular simulations through multi-level parallelism from laptops to supercomputers. SoftwareX 2015, 1–2, 19–25. 10.1016/j.softx.2015.06.001.

[ref111] Abraham M.; Alekseenko A.; Basov V.; Bergh C.; Briand E.; Brown A.; Doijade M.; Fiorin G.; Fleischmann S.; Gorelov S. GROMACS 2024.1 Manual, 2024 (accessed on 04-10-2024).

[ref112] Monticelli L.; Kandasamy S. K.; Periole X.; Larson R. G.; Tieleman D. P.; Marrink S.-J. The MARTINI coarse-grained force field: extension to proteins. J. Chem. Theory Comput. 2008, 4, 819–834. 10.1021/ct700324x. 26621095

[ref113] Adjoua O.; Lagardère L.; Jolly L.-H.; Durocher A.; Very T.; Dupays I.; Wang Z.; Inizan T. J.; Célerse F.; Ren P.; et al. Tinker-HP: Accelerating molecular dynamics simulations of large complex systems with advanced point dipole polarizable force fields using GPUs and multi-GPU systems. J. Chem. Theory Comput. 2021, 17, 2034–2053. 10.1021/acs.jctc.0c01164. 33755446 PMC8047816

[ref114] Cardenas A. E.; Hunter A.; Wang H.; Elber R. ScMiles2: A script to conduct and analyze Milestoning trajectories for long time dynamics. J. Chem. Theory Comput. 2022, 18, 6952–6965. 10.1021/acs.jctc.2c00708. 36191005 PMC10336853

