# Supplementary material for: Expanded Functionality and Portability for the Colvars Library
Source: J Phys Chem B. 2024 Nov 6;128(45):11108–23. doi: 10.1021/acs.jpcb.4c05604 (PMC11572706; doi:10.1021/acs.jpcb.4c05604)
Supplement: Supplementary file 1 — jp4c05604_si_001.pdf [file jp4c05604_si_001.pdf]

Supporting Information

October 8, 2024

1 Computational details

1.1 Alchemical decoupling of ketoprofen from water

CGENFF parameters for ketoprofen were produced using the Ligand reader feature[1] of CHARMM-GUI[2]. Ketoprofen was solvated in a 25-Åcubic periodic box of TIP3P water molecules using the Solvate plugin of VMD. Simulations were run using NAMD3[3] compiled with the development branch of Colvars (`https://github.com/Colvars/colvars/pull/649`) that implements lambda dynamics for the NAMD/Colvars interface. The Langevin thermostat was used to maintain a temperature of 300 K, and the Nosé-Hoover Langevin piston to maintain a pressure of 1 bar. The alchemical feature of NAMD was enabled in TI mode, decoupling electrostatic interactions for λ values between 0 and 0.5, then Lennard-Jones interactions between 0.5 and 1.0. The soft-core shift coefficient was set to 6 Å^2. In Colvars, an alchemical λ parameter was defined with a grid between 0 and 1, in steps of 0.01. Overdamped Langevin dynamics on the extended parameter λ was enabled with a fictitious mass of 150000 fs^2.kcal/mol, a target temperature of 300 K, and a damping parameter of 1000 ps$^-$1. ABF was applied to this variable, with a `fullSamples` parameter of 500 steps. 4 replicas were run concurrently, and shared ABF was enabled, sharing free-energy gradient estimates between replicas every 1000 steps.

1.2 Orientation of an axially asymmetric membrane protein

The atomic structure of rhodopsin (PDB ID: 1F88) was downloaded in the orientation predicted by the positioning of proteins in membrane (PPM) method [4], as available in online databases [5, 6]. This structure was embedded in a pre-equilibrated symmetric 200-lipid POPC bilayer. All molecules were described under the MARTINI 2.2 force field [7], and the protein's tertiary structure was fixed via elastic bonds with a force constant of 500 kJ/mol/nm^2.

MD simulations were carried out with GROMACS 2024, using an integration step of 20 fs, a nonbonded cutoff distance of 1.2 nm, a 300 K temperature maintained by velocity rescaling[8] and 1 atm pressure by the Parrinello-Rahman method [9, 10, 11]. Lateral diffusion of the protein in the dimensions parallel to the bilayer was prevented by a 2.5 kJ/mol/nm^2 restraint on a `distanceXY` variable defined between the protein's and the bilayer's initial centers of mass. Rotational diffusion of the protein was similarly prevented by a 0.28 kJ/mol/degree^2 restraint on the `spinAngle` variable that quantifies the magnitude of rotation around the bilayer's normal axis. The latter restraint is orthogonal by definition to atomic forces acting along tilt rotations, which measure the magnitude of rotations away from the normal axis, a fact that was also confirmed by numerical results. A conventional MD simulation of 20 μs was followed by metadynamics for the same simulated duration, with Gaussian hills of height 0.002 kJ/mol and half-width σ = 0.001 added every 1000 steps.

2 Gradients of colvar components based on a roto-translational fit

2.1 Notations

ζ	fitted colvar component
f	unfitted colvar component
$\phi = \phi_{\mathbf{X}^{(\text{fit})}}$	roto-translational fit
N	number of relevant atoms to the cvc
n	number of atoms in main group
$n^{(\text{fit})}$	number of atoms in fitting group
$\mathbf{X}$	all relevant Cartesian coords (main + fit group)
$\mathbf{X}^{(\text{fit})}$	fit group Cartesian coords
$\bar{\mathbf{x}}^{(\text{fit})}$	cog of fit group
$\mathbf{X}^{(\text{ref})}$	ref coords (constant)
$\bar{\mathbf{x}}^{(\text{ref})}$	cog of ref coords (constant)
$\mathbf{X}' = \phi(\mathbf{X})$	fitted Cartesian coords of main group
$\mathbf{x}_k$	Cartesian coords for atom k
x_k^α	Cartesian coord α (1-3) of atom k
$\partial_{k\alpha}$	partial derivative with respect to x_k^α

2.2 General case

Fitted colvar component is defined as

$$\zeta(\mathbf{X}) = f(\mathbf{X}') = f(\phi(\mathbf{X}))$$

where ϕ is the fitting function in $\mathbb{R}^3$:

$$\phi_{\mathbf{X}^{(\text{fit})}}(\mathbf{x}) = R(\mathbf{x} - \bar{\mathbf{x}}^{(\text{fit})}) + \bar{\mathbf{x}}^{(\text{ref})}$$

which depends on $\mathbf{X}^{(\text{fit})}$ explicitly through $\bar{\mathbf{x}}^{(\text{fit})}$ and implicitly because R is the optimal rotation matrix to bring $\mathbf{X}^{(\text{fit})} - \bar{\mathbf{x}}^{(\text{fit})}$ onto $\mathbf{X}^{(\text{ref})} - \bar{\mathbf{x}}^{(\text{ref})}$.

$$\partial_{k\alpha}\zeta(\mathbf{X}) = \partial_{k\alpha}f(\mathbf{X}') \tag{1}$$

$$= \sum_{l,\text{main}} \nabla_l f \cdot \partial_{k\alpha}\phi(\mathbf{x}_l) \tag{2}$$

where index l runs over atoms belonging to the main group, whose coordinates f depends on *explicitly*.

$$\partial_{k\alpha}\phi(\mathbf{x}_l) = \partial_{k\alpha}R(\mathbf{x}_l - \bar{\mathbf{x}}^{(\text{fit})}) + R(\delta_{kl}\mathbb{1}_\alpha - \partial_{k\alpha}\bar{\mathbf{x}}^{(\text{fit})}) \tag{3}$$

where $\mathbb{1}_\alpha$ is shorthand for $(\delta_{i\alpha})_i$.

The general expression of the gradient is thus:

$$\partial_{k\alpha}\zeta(\mathbf{X}) = \sum_{l,\text{main}} \nabla_l f \cdot \left[\partial_{k\alpha}R(\mathbf{x}_l - \bar{\mathbf{x}}^{(\text{fit})}) + R(\delta_{kl}\mathbb{1}_\alpha - \partial_{k\alpha}\bar{\mathbf{x}}^{(\text{fit})})\right] \tag{4}$$

For any atom k belonging to the fit group, $\partial_{k\alpha}\bar{\mathbf{x}}^{(\text{fit})} = \frac{1}{n^{(\text{fit})}}\mathbb{1}_\alpha$; that quantity, as well as $\partial_{k\alpha}R$, is zero for atoms not belonging to the fit group. We note 1_k^{fit} the indicator function for atom k belonging to the fit group, and 1_k^{main} for k belonging to the main group (in the most general case, both may be equal to 1). We can rewrite the previous expression in vector form:

$$\nabla_k\zeta(\mathbf{X}) = 1_k^{\text{main}}R^{-1}\nabla_k f - \frac{1_k^{\text{fit}}}{n^{(\text{fit})}}R^{-1}\sum_{l,\text{main}}\nabla_l f + \sum_{l,\text{main}}\nabla_l f \cdot \nabla_k R(\mathbf{x}_l - \bar{\mathbf{x}}^{(\text{fit})}) \tag{5}$$

where the three rhs. terms correspond respectively to explicit dependence of f on main group coordinates, dependence on fitting atoms due to translational fit, and dependence on fitting atoms due to rotational fit.

We note that this requires no assumption that the fit group and main group be identical, different, or non-overlapping.

2.3 Most common case: main group is identical to fit group

2.3.1 Roto-translation

$$\partial_{k\alpha}\zeta(\mathbf{X}) = \sum_{l,\text{main}} \nabla_l f \cdot \left[\partial_{k\alpha}R(\mathbf{x}_l - \bar{\mathbf{x}}) + R\left(\delta_{kl} - \frac{1}{n}\right)\mathbb{1}_\alpha\right] \tag{6}$$

Or in vector/tensor form:

$$\nabla_k \zeta(\mathbf{X}) = \sum_{l,\text{main}} \nabla_l f \cdot \nabla_k R(\mathbf{x}_l - \bar{\mathbf{x}}) + \sum_{l,\text{main}} \nabla_l f \cdot R\left(\delta_{kl} - \frac{1}{n}\right) \mathbb{1} \tag{7}$$

$$\nabla_k \zeta(\mathbf{X}) = R^{-1} \sum_{l,\text{main}} \left(\delta_{kl} - \frac{1}{n}\right) \nabla_l f + \sum_{l,\text{main}} \nabla_l f \cdot \nabla_k R(\mathbf{x}_l - \bar{\mathbf{x}}) \tag{8}$$

If f is translation-invariant, $\sum_{l,\text{main}} \nabla_l f = \mathbf{0}$ and the first term reduces to the back-rotated gradients of f:

$$\nabla_k \zeta(\mathbf{X}) = R^{-1} \nabla_k f + \sum_{l,\text{main}} \nabla_l f \cdot \nabla_k R(\mathbf{x}_l - \bar{\mathbf{x}}) \tag{9}$$

The second term vanishes in the RMSD case, but I can't state the necessary condition for it to vanish.

2.3.2 Translation only

If only translation is present, eq (8) reduces to:

$$\nabla_k \zeta(\mathbf{X}) = \sum_{l,\text{main}} \left(\delta_{kl} - \frac{1}{n}\right) \nabla_l f \tag{10}$$

which often reduces to $\nabla_k f$ when $\sum_{l,\text{main}} \nabla_l f = \mathbf{0}$.

2.4 Special case: main group and fit group are non-overlapping

2.4.1 Roto-translation

We recall the general form or the gradient:

$$\partial_{k\alpha} \zeta(\mathbf{X}) = \sum_{l,\text{main}} \nabla_l f \cdot \left[\partial_{k\alpha} R(\mathbf{x}_l - \bar{\mathbf{x}}^{(\text{fit})}) + R(\delta_{kl} \mathbb{1}_\alpha - \partial_{k\alpha} \bar{\mathbf{x}}^{(\text{fit})})\right] \tag{11}$$

2.4.2 Main group gradient

$$\nabla_k \zeta = R^{-1} \nabla_k f \tag{12}$$

2.4.3 Fit group gradient

$$\nabla_k \zeta(\mathbf{X}) = \sum_{l,\text{main}} \nabla_l f \cdot \nabla_k R(\mathbf{x}_l - \bar{\mathbf{x}}^{(\text{fit})}) - \frac{1}{n^{(\text{fit})}} \nabla_l f \cdot R \mathbb{1} \tag{13}$$

$$= \sum_{l,\text{main}} \nabla_l f \cdot \nabla_k R(\mathbf{x}_l - \bar{\mathbf{x}}^{(\text{fit})}) - \frac{1}{n^{(\text{fit})}} R^{-1} \sum_{l,\text{main}} \nabla_l f \tag{14}$$

Again, the second term vanishes for translation-invariant coordinates.

2.4.4 Translation only

2.4.5 Main group gradient

$$\nabla_k \zeta = \nabla_k f \tag{15}$$

2.4.6 Fit group gradient

$$\nabla_k \zeta = -\frac{1}{n^{(\text{fit})}} \sum_{l,\text{main}} \nabla_l f \tag{16}$$

which obviously vanishes for translation-invariant f.

References

[1] Seonghoon Kim, Jumin Lee, Sunhwan Jo, Charles L. Brooks, Hui Sun Lee, and Wonpil Im. Charmm-gui ligand reader and modeler for charmm force field generation of small molecules: Charmm-gui ligand reader and modeler for charmm force field generation of small molecules. *J. Comput. Chem.*, 38(21):1879–1886, May 2017.

[2] Sunhwan Jo, Taehoon Kim, Vidyashankara G. Iyer, and Wonpil Im. Charmm-gui: A web-based graphical user interface for charmm. *J. Comput. Chem.*, 29(11):1859–1865, June 2008.

[3] James Phillips, David Hardy, Julio Maia, John Stone, Joao Ribeiro, Rafael Bernardi, Ronak Buch, Giacomo Fiorin, Jérôme Hénin, Wei Jiang, Ryan McGreevy, Marcelo Cardoso dos Reis Melo, Brian Radak, Robert Skeel, Abhishek Singharoy, Yi Wang, Benoît Roux, Aleksei Aksimentiev, Zan Luthey-Schulten, Laxmikant Kale, Klaus Schulten, Christophe Chipot,

and Emad Tajkhorshid. Scalable molecular dynamics on CPU and GPU architectures with NAMD. *J. Chem. Phys.*, 153:044130, 2020.

[4] Andrei L Lomize, Irina D Pogozheva, and Henry I Mosberg. Anisotropic solvent model of the lipid bilayer. 2. energetics of insertion of small molecules, peptides, and proteins in membranes. *J. Chem. Inf. Model.*, 51(4):930–946, 2011.

[5] Mikhail A. Lomize, Irina D. Pogozheva, Hyeon Joo, Henry I. Mosberg, and Andrei L. Lomize. OPM database and PPM web server: resources for positioning of proteins in membranes. *Nucleic Acids Res.*, 40(D1):D370–D376, 09 2011.

[6] Antoniya A Aleksandrova, Edoardo Sarti, and Lucy R Forrest. Encompass: An encyclopedia of membrane proteins analyzed by structure and symmetry. *Structure*, 32(4):492–504, 2024.

[7] Luca Monticelli, Senthil K Kandasamy, Xavier Periole, Ronald G Larson, D Peter Tieleman, and Siewert-Jan Marrink. The martini coarse-grained force field: extension to proteins. *J. Chem. Theory Comput.*, 4(5):819–834, 2008.

[8] Giovanni Bussi, Davide Donadio, and Michele Parrinello. Canonical sampling through velocity rescaling. *J. Chem. Phys.*, 126(1):014101, 2007.

[9] Hans C Andersen. Molecular dynamics simulations at constant pressure and/or temperature. *J. Chem. Phys.*, 72(4):2384–2393, 1980.

[10] Michele Parrinello and Aneesur Rahman. Polymorphic transitions in single crystals: A new molecular dynamics method. *Journal of Applied physics*, 52(12):7182–7190, 1981.

[11] Shuichi Nosé and ML Klein. Constant pressure molecular dynamics for molecular systems. *Mol. Phys.*, 50(5):1055–1076, 1983.
